# Beyond Bio-Inspired Algorithms: Using Bird Landing Dynamics to Design Adaptive Parameter Control in Metaheuristics

**DOI:** 10.3390/biomimetics11090611

**Published:** 2026-08-31

**Authors:** Andrés Pérez, Broderick Crawford, Eduardo Rodriguez-Tello, Jorge Mendoza, Gino Astorga, Ricardo Soto

**Affiliations:** 1 Escuela de Ingeniería Informática, Pontificia Universidad Católica de Valparaíso, Avenida Brasil 2241, Valparaíso 2362807, Chile; 2 Cinvestav Unidad Tamaulipas, Km. 5.5 Carretera Victoria-Soto La Marina, Victoria 87130, Tamaulipas, Mexico; 3 Escuela de Ingeniería Eléctrica, Pontificia Universidad Católica de Valparaíso, Avenida Brasil 2147, Valparaíso 2362804, Chile; 4 Escuela de Negocios Internacionales, Universidad de Valparaíso, Alcalde Prieto Nieto 452, Viña del Mar 2572048, Chile

**Keywords:** bio-inspired parameter design, Logarithmic Mean Optimization, exploration-exploitation balance, bird landing dynamics, Bayesian optimization, adaptive parameter control, metaheuristics, Optuna

## Abstract

Bio-inspiration has mainly been used to represent organisms, behaviors, or natural processes in the design of metaheuristics. This study proposes a different use: drawing on a biological phenomenon to model the temporal evolution of an internal parameter in an existing algorithm. Logarithmic Mean Optimization (LMO) is adopted as a case study, focusing on β, which scales the stochastic perturbation term and regulates the balance between exploration and exploitation. Inspired by the progressive transition observed during bird landing, a normalized arctangent trajectory controlled by *m* and *k* is proposed. Both hyperparameters were tuned through Bayesian optimization using the Tree-structured Parzen Estimator (TPE) implemented in Optuna. The 23 benchmark functions were divided into 12 tuning functions and 11 independent test functions. Nine β configurations were evaluated through 31 runs per function. The Friedman test showed significant differences among the variants ($p=3.8269\times 10^{-10}$), and the proposed formulation achieved the best average rank (1.7273). Post-hoc Wilcoxon tests with Holm correction found significant differences against two of the eight alternatives. Overall, the results suggest that bio-inspiration, when used as a criterion for designing adaptive parameter-control mechanisms, can yield improvements and be considered a potential alternative in the design of new metaheuristic algorithms.

## 1. Introduction

Optimization problems arising in engineering, science, and industry are often characterized by high dimensionality, multimodality, and non-convexity, conditions under which exact methods become computationally prohibitive at scale. Metaheuristic algorithms have emerged as a practical alternative in these settings, providing high-quality approximate solutions through a structured balance between global exploration and local exploitation [1,2]. Their applicability spans continuous, binary and combinatorial formulations, as well as industrial problems [3,4,5].

Over the past three decades, bio-inspired metaheuristics, whose search mechanisms are drawn from biological, physical, or social phenomena, have become particularly widespread, with representative examples including Particle Swarm Optimization (PSO) [6], Whale Optimization Algorithm (WOA) [7], Sine Cosine Algorithm (SCA) [8], and more recently the Secretary Bird Optimization Algorithm (SBOA) [9]. Despite this diversity, all such methods share a common dependency on internal parameters that regulate the dynamics of the search process.

The performance of a metaheuristic is strongly sensitive to the values assigned to its internal parameters. Small perturbations in these values can produce substantial changes in convergence speed and solution quality, a phenomenon that has been extensively documented in the parameter control literature [10,11,12]. Three broad strategies have been proposed to address this challenge: static tuning before execution, adaptive control during search, and self-adaptive mechanisms embedded within the algorithm itself [10]. However, a fundamental question that cuts across all three strategies remains open: how should the evolution of a parameter be shaped over the course of the search process? Although the optimization community has developed effective techniques to identify suitable parameter values, including statistical calibration [13], grid search, and Bayesian Optimization [14], these approaches focus primarily on selecting configurations rather than on principled modeling of parameter trajectories. The manner in which a parameter transitions from one regime to another throughout an algorithm’s execution remains largely determined by heuristic criteria or ad hoc design decisions [10,11].

Bio-inspiration has historically addressed this challenge at the level of algorithm design: biological phenomena are translated into search operators, movement rules, and update strategies that constitute the core of a new metaheuristic [1,2]. This paradigm has been highly productive, yet it implicitly assumes that the most valuable contribution of nature lies in its behavioral repertoire rather than in the control principles that govern adaptive transitions. Biological systems, however, also exhibit finely regulated internal mechanisms that modulate behavior as conditions change. Landing maneuvers in birds provide a compelling illustration: as a bird approaches a perch, it progressively transitions from high-freedom exploratory flight to precise, controlled motion, a gradient of behavior that is not fixed but continuously adapted in response to distance, velocity, and environmental signals. This type of adaptive transition has received considerable attention in biomechanics and autonomous robotics [15,16,17,18], but its potential as a model for parameter dynamics in optimization algorithms has not been investigated.

The present work proposes a fundamentally different use of bio-inspiration: rather than designing a new metaheuristic, we employ biological phenomena to model the temporal evolution of an internal parameter in an existing algorithm. Specifically, we focus on the parameter β of the Logarithmic Mean Optimization (LMO) algorithm [19], which scales the stochastic perturbation and thereby influences the exploration–exploitation dynamics. Drawing on the kinematic profile of bird landing maneuvers, we formulate a normalized arctangent function whose shape mirrors the progressive reduction in exploratory freedom observed during approach-to-perch transitions. The two hyperparameters governing this function, the transition-location parameter *m* and the decay rate k, are automatically determined by Bayesian Optimization using the Tree-structured Parzen Estimator (TPE) implemented in Optuna [20], eliminating the need for manual tuning. The final evaluation on the held-out test functions showed significant global differences among the evaluated configurations ($\chi_{F,c}^{2}$ = 60.4364, df = 8, *p* = 3.8269 × 10^−10^, Friedman test). The proposed bio-inspired formulation achieved the best average rank (1.7273) and showed statistically significant pairwise differences against two of the eight alternative configurations after Holm correction.

LMO was selected as the case study because its original formulation [19] incorporates β as a scaling factor for the stochastic perturbation, but does not prescribe its numerical value, admissible range, or temporal evolution. This under-specified aspect makes LMO a suitable experimental platform for isolating the influence of an internal parameter and evaluating whether its dynamics can be modeled through biological inspiration without modifying the remaining search mechanisms. Thus, the choice of LMO is based on methodological suitability rather than on an assumption of superiority over other metaheuristics.

The main contributions of this work are:

1. A novel perspective on bio-inspired computing, in which biological phenomena are used not to design new search mechanisms, but to model the dynamics of internal parameters in existing metaheuristics.
2. A bio-inspired dynamic β model for LMO based on a normalized arctangent function derived from the progressive transition observed during bird landing maneuvers, providing a continuous and interpretable parameter trajectory.
3. Integration with automated hyperparameter tuning, where Bayesian Optimization is used to determine a single global pair of shape parameters $\left(m^{*},k^{*}\right)$ from acharacterised using the dimension-wiset. The selected configuration is subsequently kept fixed during evaluation on an independent test set.
4. An experimental evaluation of nine alternative β configurations on held-out benchmark functions. The proposed bio-inspired formulation achieved the best average rank, while the statistical analysis identified significant global differences among the evaluated strategies and significant pairwise differences against two of the eight alternative configurations after correction for multiple comparisons.

The remainder of this paper is organized as follows. Section 2 presents the theoretical background and the development of the proposed bio-inspired parameter-control mechanism. Section 3 describes the experimental design and analyzes the results obtained on the classical benchmark functions, including the ablation study. Section 4 provides an additional validation using the CEC 2022 benchmark suite. Section 5 examines the transferability of the proposed mechanism to the Grey Wolf Optimizer. Finally, Section 6 presents the conclusions and outlines directions for future research.

## 2. Bio-Inspired Foundations for Adaptive Parameter Dynamics in Metaheuristics

### 2.1. Bio-Inspiration in Metaheuristics

Bio-inspiration has played a fundamental role in the development of modern metaheuristics. Over the past decades, numerous optimization algorithms have been designed by transferring principles observed in biological, physical, and natural systems into computational search procedures capable of addressing complex optimization problems [1,2]. This approach has enabled the development of robust and flexible methods for problems characterized by large search spaces, multimodal objective functions, and constraints that are difficult to handle using traditional deterministic techniques. The success achieved by these metaheuristics has consolidated bio-inspiration as one of the primary sources for the design of new optimization algorithms, where the observed phenomenon is typically used to define search operators, movement rules, or update strategies that form the core of the algorithm’s structure [1,2]. Beyond algorithm design, the transfer of biological principles to artificial systems has been formalised in other domains through structured transfer frameworks, which make explicit the mapping between the observed phenomenon and its computational or physical counterpart [21].

However, biological systems do not only exhibit complex behaviors, they also rely on finely regulated internal mechanisms that progressively modulate their dynamics in response to environmental conditions. This adaptive capability has attracted increasing interest across different research areas, where efforts have been made to understand how certain biological principles can contribute to improving the efficiency and robustness of artificial systems [22]. From this perspective, bio-inspiration can be interpreted not only as a tool for designing new search mechanisms, but also as a source of principles capable of modeling the temporal evolution of internal parameters in existing metaheuristics. This distinction constitutes the conceptual foundation of the present work: rather than using biological phenomena to construct a new algorithm, they are used here to define the dynamics of a parameter that governs the adaptive behavior of an existing one.

### 2.2. Bird Landing Dynamics

Bird landing constitutes one of the most complex phases of flight due to the need to simultaneously coordinate stability, trajectory control, speed reduction, and spatial precision in order to successfully reach the point of contact [15,16,17,18,23,24]. Unlike a linear approach toward the target, birds perform continuous adjustments in direction, speed, and body posture, allowing them to respond to environmental disturbances and dynamically adapt to different landing conditions.

Several biomechanical studies have shown that these maneuvers exhibit characteristics close to optimal behavior from both energetic and dynamic perspectives [15]. During the initial stages of approach, birds possess a high degree of freedom of movement, enabling them to explore different trajectories and compensate for environmental variations. However, as the distance to the target decreases, this freedom is progressively reduced, giving rise to a phase characterized by increasingly precise trajectory control, where fine adjustments predominate in order to ensure a stable and safe landing [16,17,18,23,24].

Recent literature has further shown that birds use visual information from the environment, particularly optic flow patterns, to estimate distance, relative velocity, and the remaining time until landing [23]. This information is used to continuously adapt the approach, regulating both the intensity and frequency of the corrections required during flight. As a result, landing can be interpreted as an adaptive process in which behavior progressively evolves as uncertainty regarding the final target decreases.

A quantitative example of this phase-structured transition was reported by Ponitz et al. [25], who reconstructed the three-dimensional descent and landing trajectory of a peregrine falcon. The maneuver was divided into six phases: acceleration and diving, a transient phase at approximately constant speed, deceleration and flight-path correction, pull-out, landing at approximately constant speed, and final deceleration with touchdown. During the initial descent, the falcon covered a vertical distance of 18.55 m while increasing its speed from 15.0 to 22.5 m/s. It then entered the correction phase, during which its speed decreased to 19.4 m/s and its flight-path angle progressively declined from 50.75° to 0°. This reconstructed maneuver provides quantitative evidence that the transition toward landing involves coordinated changes in altitude, speed, acceleration, and trajectory direction.

**Figure 1:**
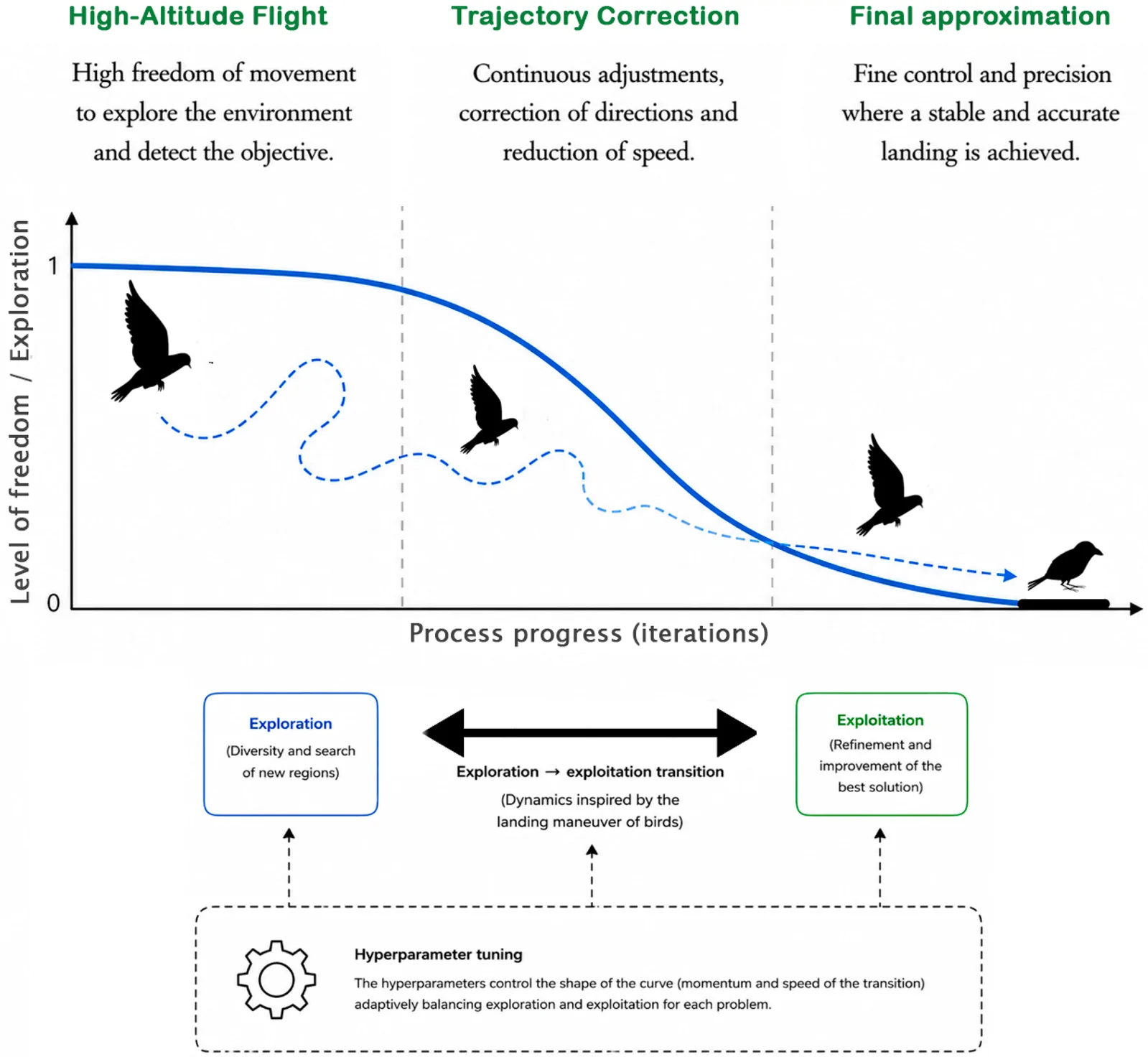
Conceptual illustration of the transition observed during bird landing maneuvers, from an initial phase of exploration and trajectory correction to a final stage of controlled and precise approach. This dynamic serves as the inspiration for the progressive regulation of the β parameter proposed in this work.

Figure 1 presents a conceptual abstraction of this phase-structured transition. It represents the progression from an initial flight regime with greater freedom of movement, through a phase of deceleration and trajectory correction, toward a controlled final approach. The figure does not reproduce a measured biological trajectory or imply that bird altitude follows an arctangent law. Instead, it transfers the observed sequence of flight phases to the progressive regulation of exploration and exploitation.

This behavior has attracted interest not only in biomechanics, but also in fields such as aerial robotics, autonomous control, and bio-inspired vehicles [16,17,18]. Several studies have demonstrated that the principles observed during landing maneuvers can be used to improve navigation strategies and trajectory planning, suggesting that nature has developed highly efficient mechanisms for regulating the transition between global behaviors and local control actions. From a biological perspective, these observations reveal that landing maneuvers do not correspond to a fixed sequence of movements, but rather to a dynamic process of continuous adaptation. The combination of freedom of movement during the initial phases and precise control during the final stages constitutes a recurring characteristic observed across different species and landing scenarios [15,16,17,18,23,24]. This adaptive capability is particularly interesting from an optimization perspective. For decades, the scientific community has developed various parameter tuning, fine-tuning, and hyperparameter optimization strategies with the aim of improving the performance of metaheuristics and promoting an appropriate search balance [10,13,20]. More recently, automated approaches such as Bayesian Optimization have enabled the identification of high-performance configurations while reducing researcher intervention [14,20,26]. However, although these techniques allow suitable parameter values to be determined, they generally focus on optimizing specific configurations rather than understanding how such parameters should evolve throughout the search process. In this context, the dynamics observed during bird landing provide a complementary perspective: using biological phenomena as a source of inspiration for modeling the temporal evolution of internal parameters responsible for the adaptive behavior of a metaheuristic.

### 2.3. Exploration–Exploitation Behavior in Benchmark Functions

Bird landing maneuvers exhibit a progressive transition from states characterized by a high degree of freedom of movement toward stages of greater control and precision, enabling a smooth landing. From an optimization perspective, this dynamic raises a relevant question: do high-performing metaheuristic algorithms exhibit similar behavior? Answering this question is particularly important for the present work, since the objective is not merely to transfer a biological analogy into an optimization algorithm, but to determine whether such an analogy can be observed in actual behavioral patterns. If high-performing algorithms exhibit a transition that is, at least descriptively, observable and consistent between exploration and exploitation, then this bio-inspired dynamic may be used to model the temporal evolution of the β parameter within Logarithmic Mean Optimization (LMO). For this purpose, an experimental analysis was conducted to characterize the exploration–exploitation behavior of different representative algorithms from the literature. In particular, Secretary Bird Optimization Algorithm (SBOA) [9], Whale Optimization Algorithm (WOA) [7], Sine Cosine Algorithm (SCA) [8], Particle Swarm Optimization (PSO) [6], Pendulum Search Algorithm (PSA) [27] and Arithmetic Optimization Algorithm (AOA) [28] were selected. The selection includes algorithms inspired by different search principles, allowing the identification of common trends associated with superior performance. The experiments were carried out using the 23 classical benchmark functions commonly employed in the evaluation of metaheuristics. Each algorithm was executed for 100 iterations using a population of 50 individuals and a total of 31 independent runs per function.

Search behaviour was characterised using the dimension-wise population diversity measure of Cheng et al. [29], given by

(1) $$\mathrm{Div}\left(t\right)=\frac{1}{D\;N}\sum_{j=1}^{D}\sum_{i=1}^{N}\left|{\bar{x}}_{j}\left(t\right)-x_{i,j}\left(t\right)\right|,\;{\bar{x}}_{j}\left(t\right)=\frac{1}{N}\sum_{i=1}^{N}x_{i,j}\left(t\right),$$

where *N* is the population size, *D* the problem dimension, $x_{i,j}\left(t\right)$ the *j*-th coordinate of individual *i* at iteration *t*, and ${\bar{x}}_{j}\left(t\right)$ the mean of that coordinate over the population. Hussain et al. [30] later proposed a variant of this measure in which the mean is replaced by the median; the mean-based formulation of [29] is used here.

The normalised exploration and exploitation indicators were then obtained following [30]:

(2) $$\mathrm{XPL}\left(t\right)=\frac{\mathrm{Div}(t)}{{\mathrm{Div}}_{\max}}\times 100,\;\mathrm{XPT}\left(t\right)=\frac{\left|\mathrm{Div}\left(t\right)-{\mathrm{Div}}_{\max}\right|}{{\mathrm{Div}}_{\max}}\times 100,$$

where ${\mathrm{Div}}_{\max}$ is the largest diversity observed up to iteration *t* within the run, initialised with the diversity of the initial population. Since $\mathrm{Div}\left(t\right)\leq {\mathrm{Div}}_{\max}$ by construction, the two indicators satisfy XPT(t)=100−XPL(t).

The values reported in Table 1 are the mean values of these indicators across iterations, independent runs, and benchmark functions. They therefore express the relative degree of population dispersion or concentration, and should not be read as the proportion of execution time or the percentage of iterations devoted to each search behaviour.

Based on these executions, the observed mean values of the normalized exploration and exploitation indicators were obtained to identify behavioral patterns that could subsequently be compared with the dynamics observed during bird landing maneuvers and used as the basis for constructing the proposed model for the β parameter.

**Figure 2:**
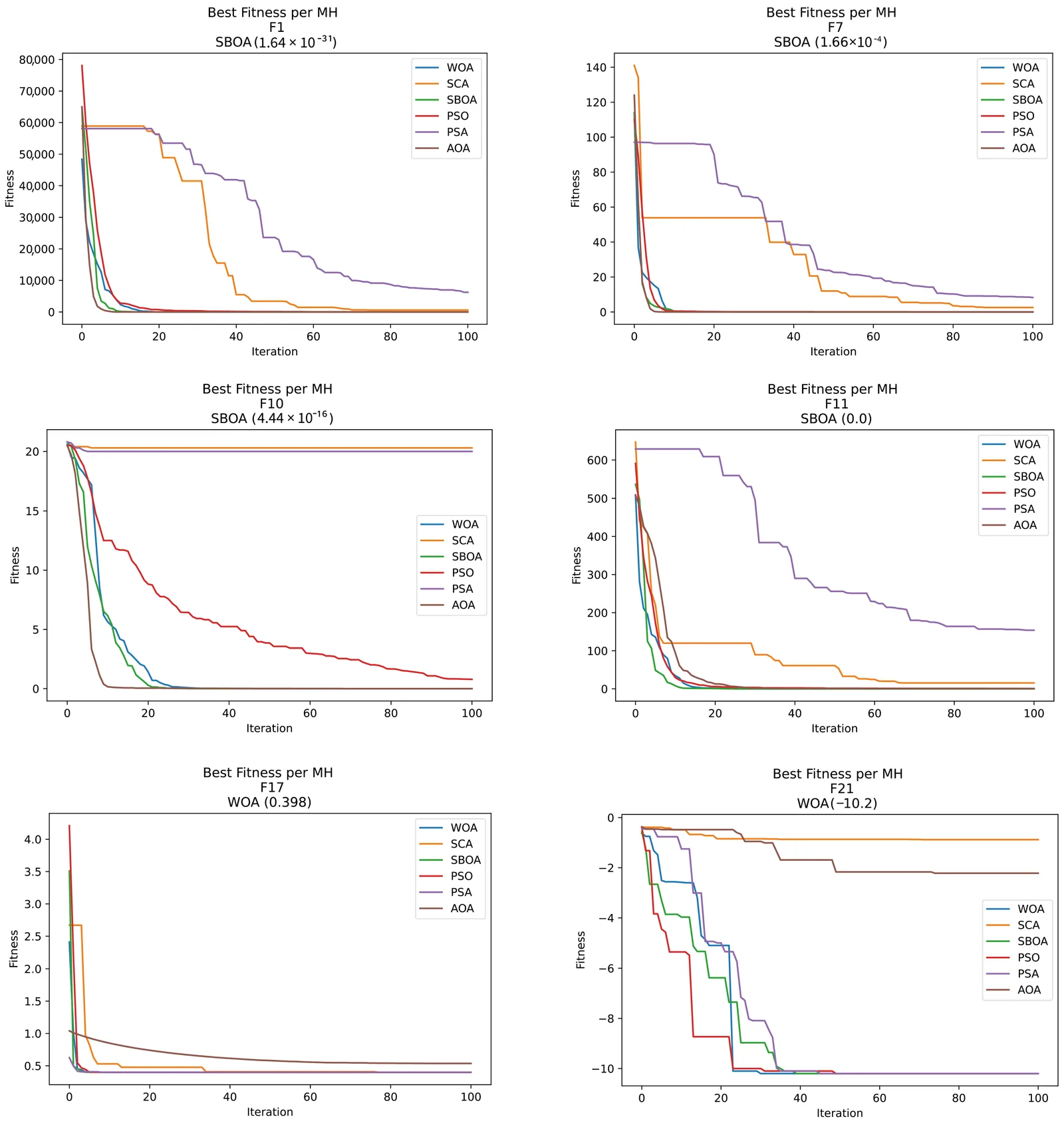
Convergence curves and fitness evaluations obtained for different benchmark functions using the experimental algorithms.

The obtained results (Figure 2) indicate the existence of a relationship between the performance of a metaheuristic and the manner in which it distributes its exploration and exploitation stages.

In particular, algorithms such as SBOA, PSO, and WOA, which exhibit faster convergence and better performance on the evaluated benchmark functions, show a marked reduction in the normalized exploration indicator during the early iterations, followed by the dominance of the exploitation indicator as the population concentrates around promising regions. Descriptively, the observed mean values of the normalized exploration indicator were generally in the 10–20% range for these algorithms, although values approaching 30% were also observed among other evaluated configurations. In contrast, lower-performing algorithms, such as SCA, maintain higher exploration indicator values for a longer period of the search process. These observations indicate that a relatively early transition from exploration to exploitation may constitute a characteristic associated with superior performance, providing an initial reference for the modeling and bio-inspired design of the β parameter.

The results summarized in Table 1 show that the best-performing algorithms share similar patterns in the observed values of their normalized exploration and exploitation indicators. In particular, PSO, SBOA, WOA, and PSA exhibit considerably lower observed mean values of the exploration indicator than SCA, together with correspondingly higher exploitation indicator values. This trend is consistent with the convergence curves observed previously, where these algorithms reach high-quality solutions during the early iterations and subsequently refine the most promising regions of the search space.

From these observations, it is possible to identify three recurring characteristics associated with the best-performing algorithms. First, exploration is concentrated primarily during the early stages of the search process. Second, the transition toward exploitation occurs relatively early, avoiding prolonged periods of random exploration. Finally, the most competitive algorithms exhibit accelerated convergence during the first third of the available iterations, followed by a more stable refinement phase.

These results provide an initial reference for the design of the β parameter proposed in this work. Rather than defining a fixed value, the observations support the convenience of a dynamic mechanism capable of promoting exploration during the initial iterations and progressively reducing it as the search process advances. This characteristic also presents a remarkable similarity to the transition observed during the bird landing maneuvers described in the previous section.

It is worth noting that this trend is consistent with observations previously reported in the literature. Mirjalili [8] points out that excessively prolonged exploration can negatively affect the efficiency of metaheuristic processes by delaying the exploitation of promising regions within the search space. The results obtained for SCA exhibit behavior consistent with this observation, reinforcing the importance of properly regulating the balance between exploration and exploitation.

### 2.4. Influence of the β Parameter on the Search Dynamics of LMO

The analysis conducted in the previous section showed that the best-performing algorithms exhibit a relatively early transition from exploration to exploitation. However, in order to transfer this observation to the Logarithmic Mean Optimization (LMO) algorithm, it is necessary to understand the effect exerted by the parameter β on the search dynamics.

In the original LMO formulation [19], the parameter β appears in the candidate-generation rule as a scaling factor for the stochastic perturbation:

(3) $$x_{\mathrm{new}}=L\left(x_{i},x_{j}\right)+\beta \left(\mathrm{rand}()-0.5\right)\left(\mathrm{ub}-\mathrm{lb}\right),$$

where $L(x_{i},x_{j})$ denotes the logarithmic-mean operator and lb and ub are the lower and upper bounds of the search space, respectively. In this expression, β scales the magnitude of the random displacement around the logarithmic-mean solution. Therefore, larger values of β allow potentially wider stochastic movements, whereas smaller values restrict the perturbation to a narrower region.

The original LMO study identifies β as a control parameter but does not provide a numerical value, an admissible range, or a time-dependent rule for its evolution. Moreover, LMO incorporates an independent adaptive parameter, $\alpha_{t}$, which progressively increases the influence of the best solution:

(4) $$x_{\mathrm{final}}=\left(1-\alpha_{t}\right)x_{\mathrm{new}}+\alpha_{t}x_{\mathrm{best}},\;\alpha_{t}=\frac{t}{{\mathrm{Iter}}_{\max}}.$$

Consequently, β should not be interpreted as the sole controller of the exploration–exploitation balance. Instead, it modulates the stochastic component of the LMO update rule and therefore influences the search dynamics. Since the original formulation does not specify how β should be selected or varied, its effect was empirically investigated in this work using several fixed values while keeping the remaining components of LMO unchanged.

For this purpose, an experimental study was conducted using five variants of LMO, keeping the same base implementation of LMO across all configurations and modifying only the value of β. The evaluated configurations were:

LMOB1 (β = 1), LMOB075 (β = 0.75), LMOB05 (β = 0.5), LMOB025 (β = 0.25), LMOB01 (β = 0.1).

In this way, it was possible to isolate the effect of β and analyze how different levels of this parameter affect the exploration and exploitation behavior of the algorithm.

**Figure 3:**
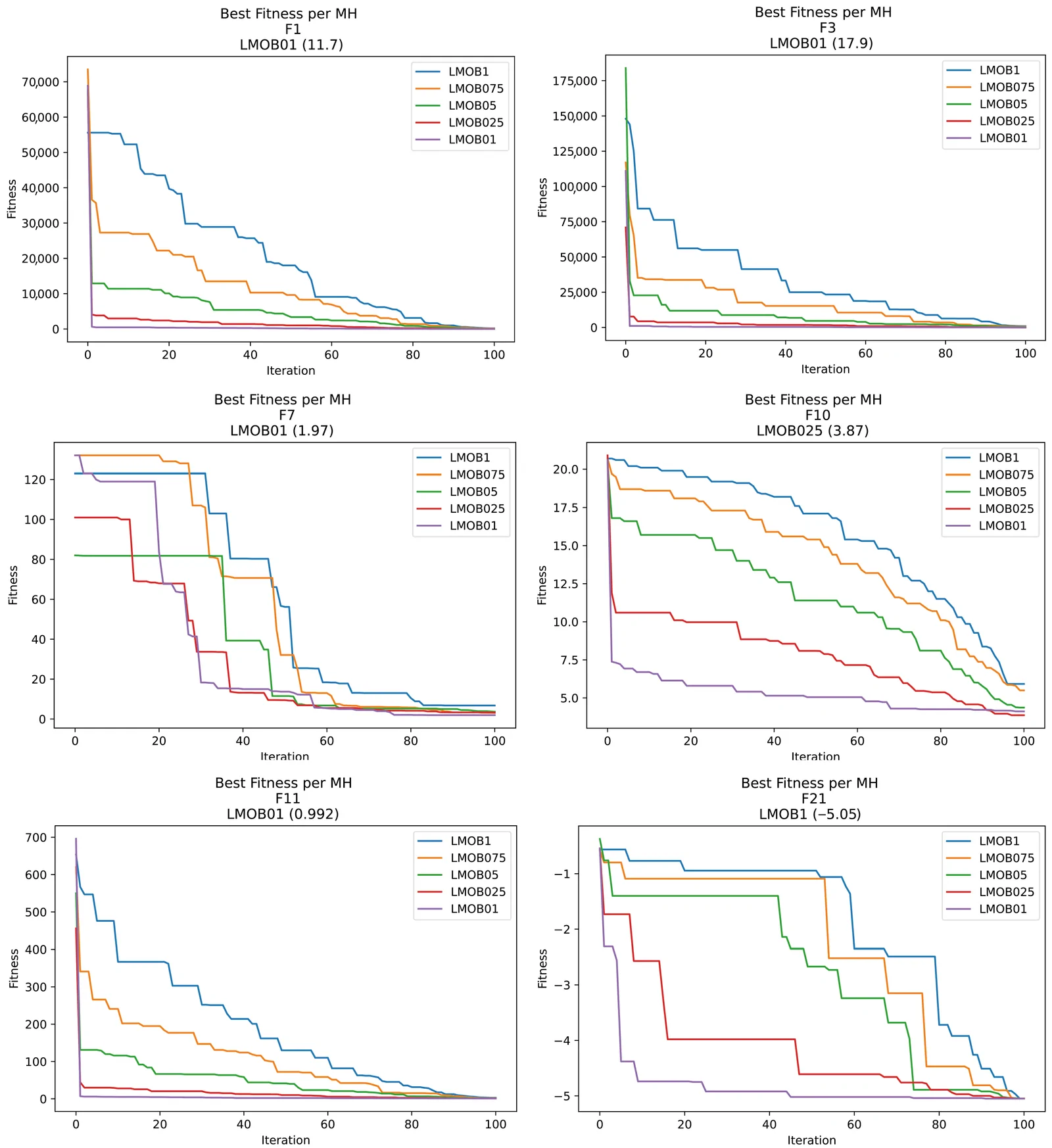
Convergence curves and fitness results obtained for LMO using different fixed values of the β parameter.

Figure 3 shows the convergence curves obtained for the different evaluated configurations. It can be observed that high values of β produce slower convergence and a lower approach toward high-quality solutions during the early stages of the search process. This behavior indicates that a significant portion of the computational effort remains devoted to exploration, hindering the early concentration of the search in promising regions of the solution space.

In contrast, configurations associated with low values of β exhibit a faster reduction in fitness and an earlier stabilization of the convergence curves. Under these conditions, the algorithm identifies favorable regions more rapidly and allocates a larger number of iterations to refining the solutions found. As a result, a more intensive exploitation process and a more consistent convergence throughout the optimization process can be observed.

Additionally, the results indicate that β not only influences the convergence speed, but also the temporal distribution of the search stages. While high values prolong exploration during a significant portion of the execution, lower values promote an earlier transition toward exploitation processes. This difference demonstrates that β plays a central role in regulating the internal dynamics of LMO and the way in which the algorithm balances both phases.

This observation is consistent with the patterns previously identified in the best-performing benchmark algorithms. In both cases, a relatively early transition toward exploitation enables competitive solutions to be reached in a smaller number of iterations, avoiding excessively prolonged search processes. Consequently, the results suggest that β constitutes an appropriate mechanism for dynamically regulating the evolution of the search process within LMO, providing a solid experimental basis for the development of the proposed bio-inspired model.

**Figure 4:**
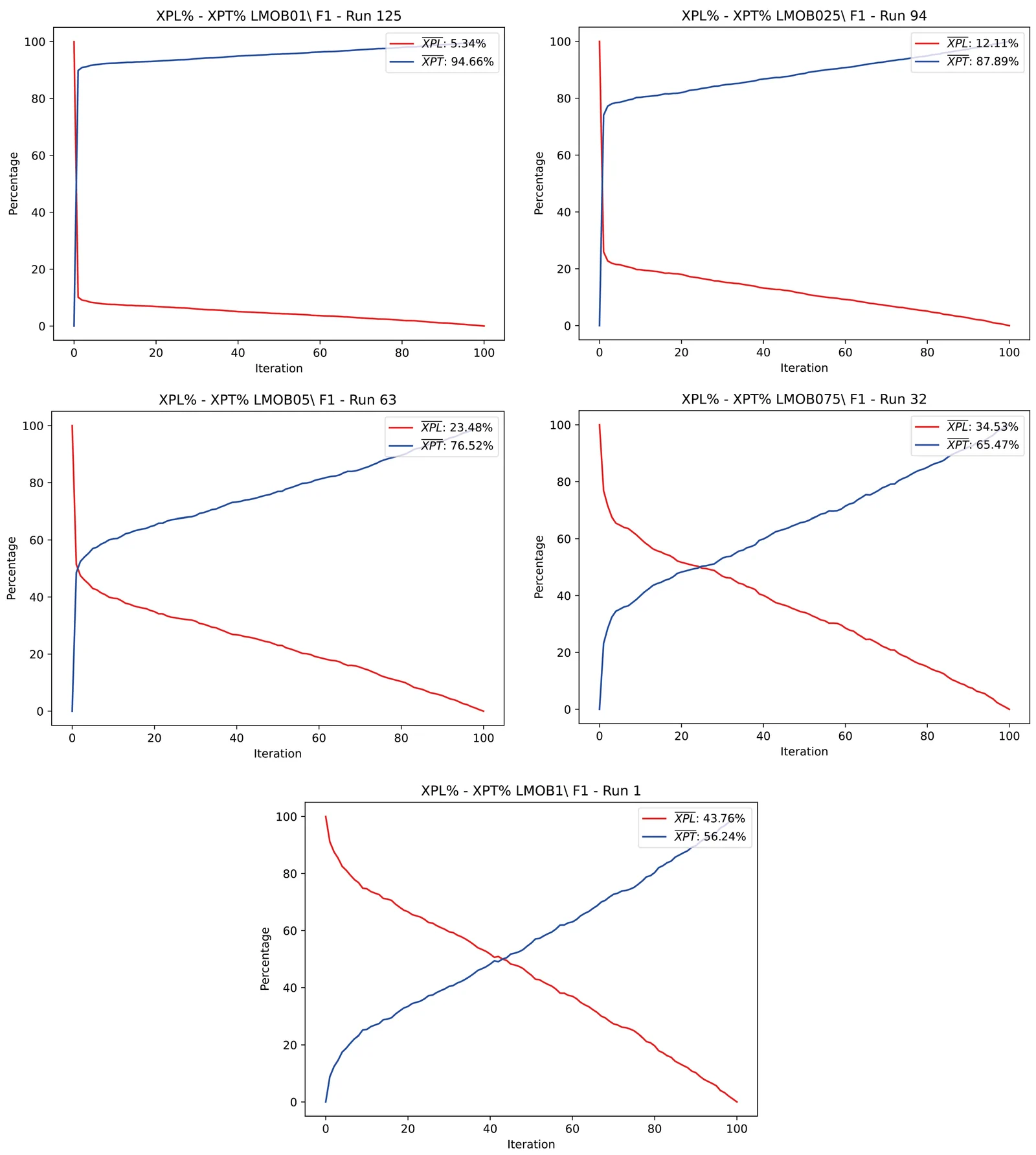
Exploration and exploitation curves obtained for LMO using different fixed values of the β parameter: β = 0.1, β = 0.25, β = 0.5, β = 0.75, and β = 1.

The exploration and exploitation behavior associated with different values of β can be observed in Figure 4, which shows that this parameter not only modifies the proportion present during the search process, but also the point at which the transition between both phases occurs.

Low values of β (0.1 and 0.25) exhibit reduced exploration percentages and a rapid dominance of exploitation mechanisms. Under these configurations, the algorithm concentrates a large portion of its efforts on refining promising solutions from the early iterations onward, a behavior consistent with that previously observed in the best-performing benchmark algorithms. As a consequence, convergence occurs earlier and a more effective exploitation of favorable regions within the search space is achieved. In contrast, high values of β (0.5, 0.75, and 1) prolong the exploratory phase throughout a considerable portion of the execution. In these cases, the transition toward exploitation occurs later, maintaining high levels of exploration during the initial iterations. This behavior results in slower convergence and a less efficient use of the available computational budget, since a significant portion of the search remains devoted to exploration even after promising regions have already been identified. A particularly relevant aspect is that variations in β produce progressive changes in the shape of the observed curves. As the value of β increases, the transition between exploration and exploitation shifts toward later iterations. This suggests that β acts as a regulatory mechanism capable of controlling not only the intensity of exploration, but also the rate at which the algorithm evolves from a global search toward a more intensive and localized search. In summary, the experimental observations establish a clear relationship between the value of β and the search dynamics of LMO:

- High β (0.5 or greater) → prolonged exploration and delayed transition toward exploitation.
- Low β (0.1 and 0.25) → early exploitation and faster convergence.

These results are consistent with observations previously reported in the literature. Mirjalili [8] points out that excessively prolonged exploration may lead to inefficient search processes, a situation that can be observed in the configurations associated with high values of β. Likewise, Eiben et al. [10] emphasize that dynamic parameter adaptation constitutes an important factor for improving the performance of evolutionary algorithms, allowing search behavior to be adjusted according to the different stages of the optimization process.

Taken together, the obtained results indicate that β should not remain constant throughout the entire execution of the algorithm. On the contrary, the results indicate the convenience of using a mechanism capable of dynamically modifying its value over the course of the iterations, promoting exploration during the early stages and exploitation as the search approaches promising regions. This conclusion constitutes the main foundation for the bio-inspired model based on bird landing proposed in this work.

### 2.5. Bio-Inspired and Experimental Principles for the Modeling of β

The observations presented in the previous sections made it possible to analyze the influence of the β parameter on the search dynamics of LMO. At the same time, the study of bird landing maneuvers and the analysis of high-performing metaheuristics revealed consistent patterns in the transition from global exploration to local refinement. Taken together, these observations provide the biological and experimental foundation supporting the principles used for the modeling of β.

From a biological perspective, the analyzed studies show that birds do not maintain a constant behavior throughout the landing maneuver. Instead, the approach toward the target is characterized by a progressive transition from states of high freedom of movement to stages dominated by mechanisms of fine control and precision [15,16,17,18,23,24]. During the initial phases, there is a greater capacity to correct trajectories and adapt to external disturbances, whereas the final stages concentrate efforts on reaching the landing point in a stable and efficient manner. This gradual transition constitutes one of the central elements of the biological inspiration employed in this work.

Consistent with these observations, the analysis conducted on benchmark algorithms showed that the best-performing metaheuristics concentrate a large portion of their exploration during the early iterations and subsequently devote most of the search process to the exploitation of promising regions. In contrast, algorithms that maintain prolonged exploration processes tend to exhibit slower convergence and a less efficient use of the available computational budget.

Additionally, the experiments conducted on LMO showed that the value of β influences this transition. High values favor prolonged exploration and delay exploitation, whereas low values promote earlier convergence and more intensive exploitation. However, the results also indicate that using a fixed value throughout the entire execution limits the algorithm’s ability to adapt to the different stages of the search process.

Based on the biological and experimental observations described above, it is possible to identify a set of principles that will serve as the foundation for the modeling of β. These principles do not represent specific values of the parameter, but rather desirable characteristics of the search dynamics that are intended to be reproduced within LMO.

Table 2 summarizes the correspondence established between the observations made during bird landing maneuvers and the patterns identified in the optimization experiments.

The principles observed during landing maneuvers exhibit a direct correspondence with the exploration and exploitation patterns identified both in the best-performing metaheuristics and in the behavior of the β parameter within LMO. This correspondence supports that the dynamics observed in the biological phenomenon constitute a valid reference for guiding the design of control mechanisms in optimization algorithms. A particularly relevant aspect is that most of the identified principles cannot be adequately represented by a fixed value of β. The high degree of freedom of movement observed during the initial phases of landing, the progressive reduction of this freedom as the bird approaches the target, and the final predominance of fine control mechanisms describe an inherently evolving dynamic. Similarly, the experimental results show that the transition between exploration and exploitation constitutes a process that unfolds throughout the execution of the algorithm rather than a static condition determined at the beginning of the search. From this perspective, bio-inspiration provides an additional element to the modeling process. While the conducted experiments make it possible to identify which types of behavior favor superior performance, the observation of birds provides a reference for how such a transition should occur. Consequently, the biological phenomenon is not used solely as a conceptual analogy, but as a source of principles capable of guiding the formulation of a dynamic mechanism for β. This reasoning constitutes the main foundation of the mathematical model developed in the following section.

### 2.6. Formulation of a Bio-Inspired Equation for β

The principles defined in the previous section make it possible to establish the characteristics that the function responsible for modeling β must satisfy. In particular, this function should promote a high exploratory capacity during the initial iterations, progressively reduce this behavior as the search advances, and allow control over both the timing and the speed of the transition toward exploitation. These properties are consistent with the patterns observed in the best-performing metaheuristics and with the need to avoid prolonged periods of exploration that may affect algorithm convergence.

Additionally, the transition should not occur abruptly. The observations made on bird landing maneuvers show that freedom of movement gradually decreases as the bird approaches its target, moving from a phase of exploration and trajectory correction toward a final stage dominated by fine control and precision mechanisms. Analogously, the experimental results obtained for LMO show that the search process benefits from a progressive reduction in exploration and a gradual increase in exploitation.

**Figure 5:**
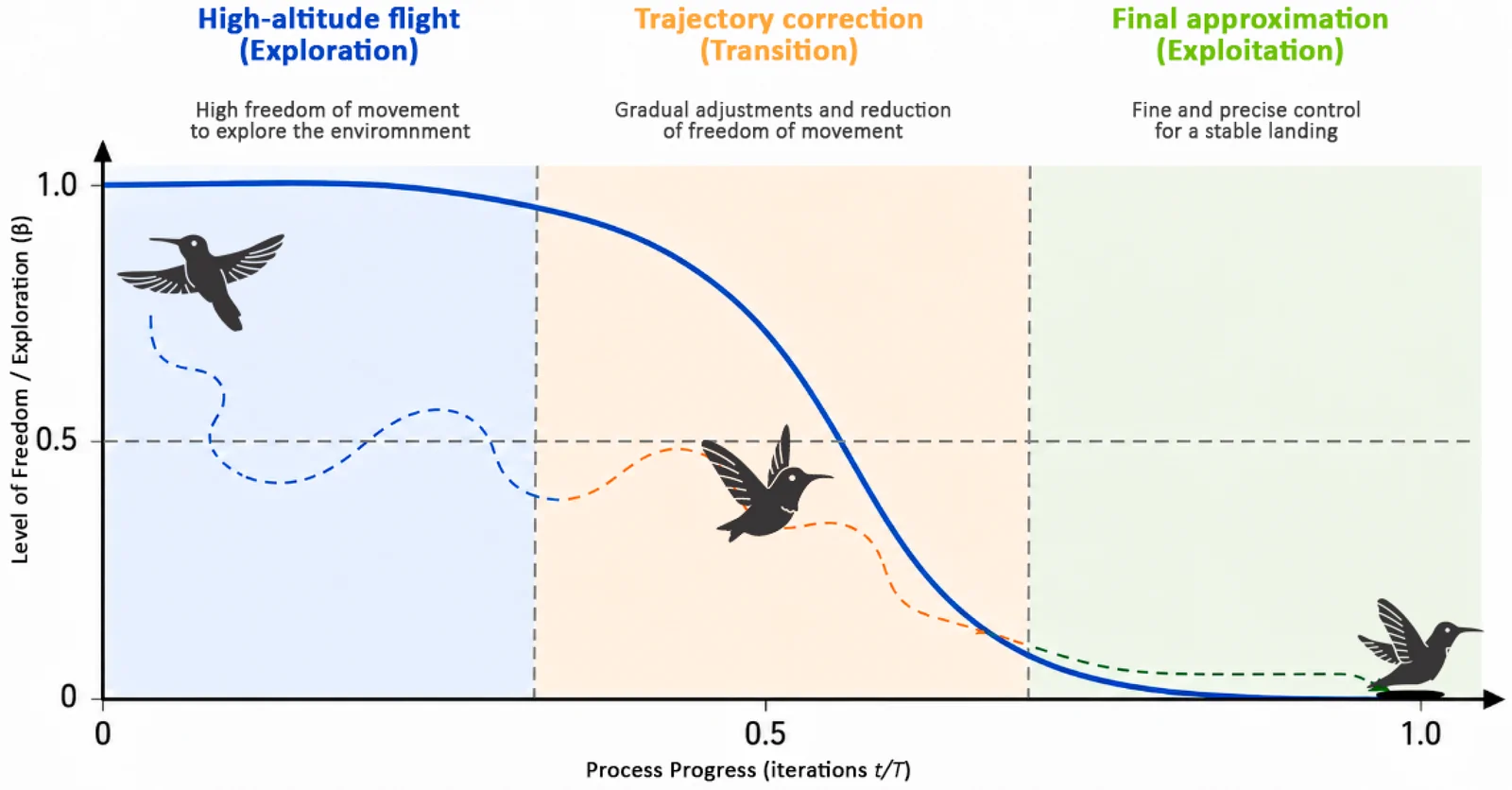
Analogy between bird flight and landing dynamics and the concepts of exploration and exploitation.

Figure 5 illustrates this conceptual analogy. It shows how landing dynamics can be interpreted as a continuous transition from an initial phase of high freedom of movement, associated with exploration, toward a final phase of controlled approach, associated with exploitation. This evolution suggests the use of a continuous, smooth, and parameterizable function capable of representing different transition profiles and reproducing the behavior observed both in the biological phenomenon and in the experimental results analyzed previously.

**Figure 6:**
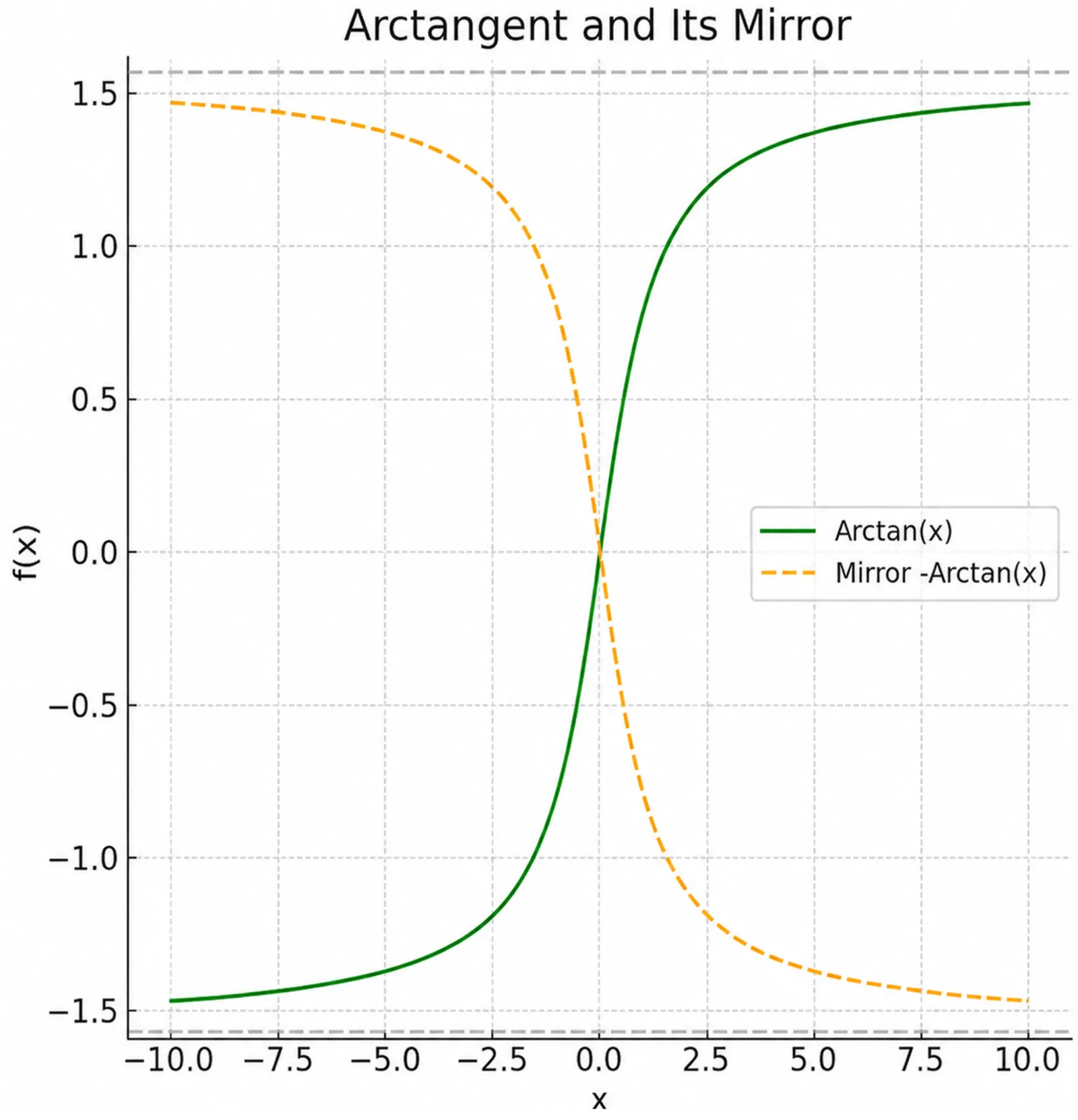
Arctangent function and its reflected form.

The phase structure observed during the descent and landing maneuver motivates the temporal organization of the proposed model rather than prescribing a unique mathematical function. In this work, a normalized arctangent was selected as a parsimonious mathematical representation of this transition. Its smooth and monotonic profile can represent an initial regime of relatively high movement freedom, a principal region of change, and a final regime associated with a controlled approach. Moreover, its parameters allow the temporal location and steepness of the transition to be regulated independently. The proposed formulation does not imply that bird landing trajectories exactly follow an arctangent law or that the arctangent is the only function capable of representing this behavior. Instead, it constitutes the bio-inspired modeling hypothesis evaluated in this study. Figure 6 shows the general shape of the selected function.

In order to reproduce the progressive transition observed during landing maneuvers, the proposed function must satisfy two fundamental conditions. First, β must remain bounded within the interval [0, 1], allowing high values to be interpreted as a greater tendency toward exploration and low values as a predominance of exploitation. Second, the function must allow control over the moment at which the transition between both phases occurs, so that it can be adapted to different optimization scenarios.

The auxiliary transition function is defined as

(5) $$g\left(t\right)=\arctan\left(k(m-t)\right),$$

where *t* denotes the current iteration, *m* controls the temporal location of the transition, and k>0 controls its steepness. Larger values of *k* produce a more abrupt transition, whereas smaller values produce a smoother change.

To ensure that the parameter remains bounded within the interval [0,1], the auxiliary function is normalized using its values at the endpoints of the iteration domain:

$$g_{0}=g\left(0\right)=\arctan\left(k(m-0)\right),$$

and

$$g_{100}=g\left(100\right)=\arctan\left(k(m-100)\right).$$

The normalized dynamic parameter used throughout the optimization process is then defined as

(6) $$\beta \left(t\right)=\frac{g\left(t\right)-g_{100}}{g_{0}-g_{100}}.$$

At each iteration *t*, the value of β(t) obtained from Equation (6) replaces the unspecified parameter β in the stochastic perturbation term of the original LMO candidate-generation rule given in Equation (3). All remaining components of LMO are kept unchanged.

Thus, Equation (5) defines the auxiliary arctangent function, whereas Equation (6) defines the normalized trajectory of the dynamic parameter β.

Considering t∈[0,100] as the iteration domain, the endpoint conditions of the normalized formulation follow directly from Equation (6). At the initial iteration,

$$\begin{array}{cc} \beta (0)\; & =\frac{g\left(0\right)-g_{100}}{g_{0}-g_{100}}\\ \; & =\frac{g_{0}-g_{100}}{g_{0}-g_{100}}\\ \; & =1. \end{array}$$

Similarly, at the final iteration,

$$\begin{array}{cc} \beta (100)\; & =\frac{g\left(100\right)-g_{100}}{g_{0}-g_{100}}\\ \; & =\frac{g_{100}-g_{100}}{g_{0}-g_{100}}\\ \; & =0. \end{array}$$

Therefore, the normalization guarantees that β(t) starts at 1, corresponding to the highest influence of exploration, and ends at 0, corresponding to the highest influence of exploitation. The remaining parameters control the shape of the transition:

- *m* controls the temporal location of the main transition from high to low values of β.
- k>0 controls the steepness of the transition. Larger values of *k* produce a more abrupt decay, whereas smaller values produce a smoother and more gradual transition.

**Figure 7:**
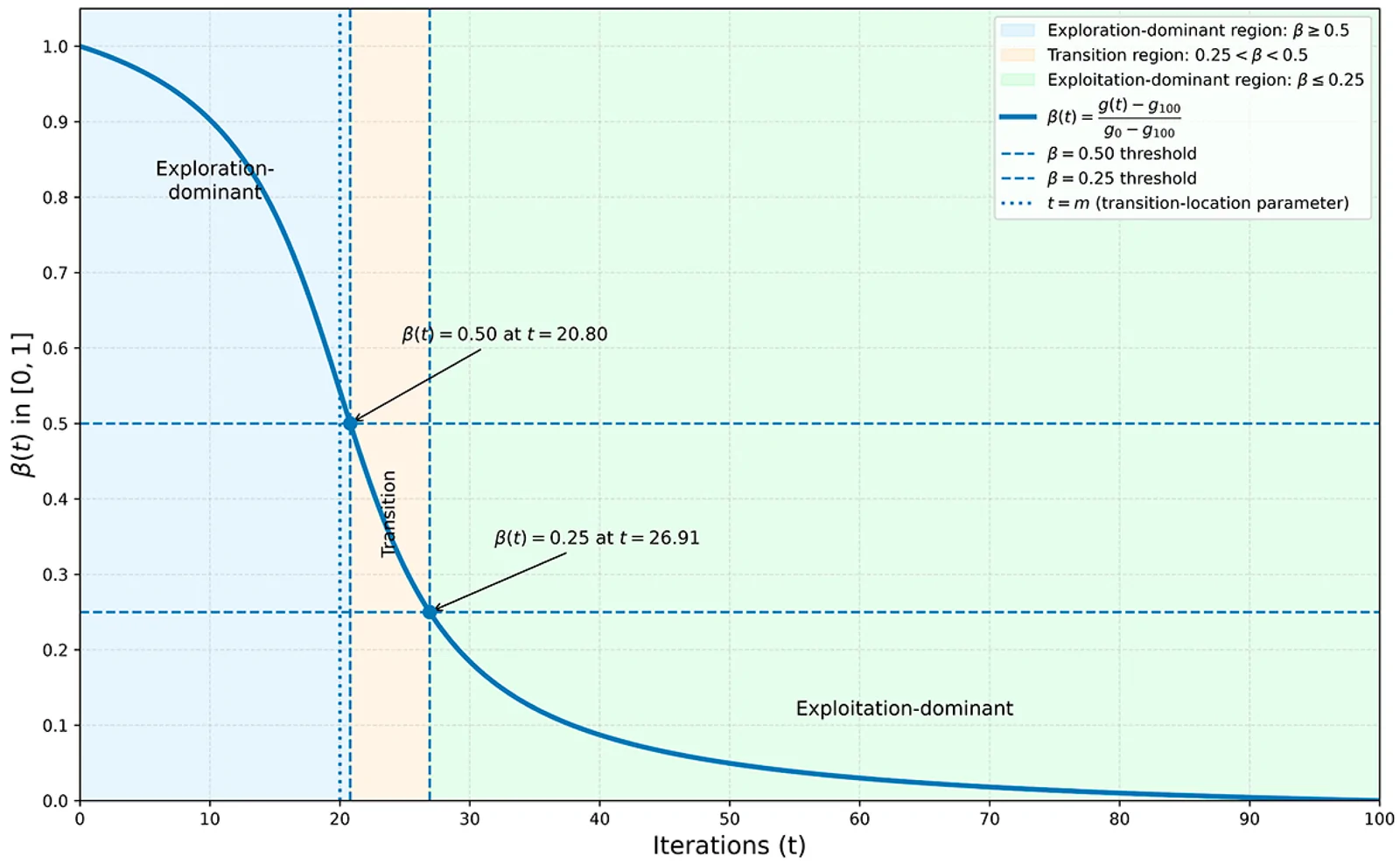
Illustrative dynamic β(t) trajectory generated from the proposed normalized arctangent formulation using m=20 and k=0.15. These parameter values are used only to illustrate the shape and temporal behavior of the proposed trajectory and do not correspond to the globally tuned configuration employed in the final experiments. For visualization purposes, the shaded regions indicate an exploration-dominant region (β≥0.5), a transition region (0.25<β<0.5), and an exploitation-dominant region (β≤0.25). These thresholds are illustrative and should not be interpreted as strict boundaries between exploration and exploitation. The vertical dotted line indicates the transition-location parameter *m*, whereas the horizontal dashed lines indicate the illustrative thresholds β=0.5 and β=0.25.

Figure 7 shows that the proposed formulation is capable of reproducing a progressive transition from high values of β toward values close to zero while maintaining a continuous behavior free from abrupt changes. During the early iterations, high values of β predominate, favoring the exploration of the search space, whereas the gradual decrease of the curve induces a progressive increase in exploitation as the optimization process advances.

The resulting equation is grounded in the observations made on bird landing maneuvers and in the patterns identified in the best-performing metaheuristics analyzed previously. Its formulation enables a controlled regulation of the influence of the parameter β on the balance between global and local search, reproducing a gradual transition from states characterized by a high degree of freedom of movement toward stages dominated by more precise control mechanisms. From this perspective, the proposed function constitutes a mathematical representation of the bio-inspired principles defined earlier and provides the foundation for the dynamic regulation of the β parameter within the LMO algorithm.

### 2.7. Bayesian Optimization Procedure

The proposed bio-inspired formulation of the parameter β represents a progressive transition from global to local search through the hyperparameters *m* and *k*. Although biological inspiration provides a coherent framework for modeling this transition, it does not uniquely determine the values that these hyperparameters should adopt. Consequently, the effectiveness of the model also depends on the appropriate selection of the hyperparameters that control the temporal position and steepness of the transition in the bio-inspired curve. This issue is not exclusive to the proposed model, as several studies have shown that the performance of optimization and machine learning algorithms can be strongly influenced by their hyperparameter configurations, with even small variations potentially producing substantial differences in the quality of the solutions obtained [31,32,33].

The manual search for suitable configurations is often computationally expensive, difficult to reproduce, and highly dependent on the researcher’s experience. As an alternative, Bayesian Optimization has emerged as an effective strategy for the automatic tuning of hyperparameters in problems where each evaluation entails a high computational cost [14,26]. Unlike traditional methods such as Grid Search and Random Search, Bayesian Optimization uses the information collected from previous evaluations to construct a probabilistic model of the search space and select new configurations with a high probability of improving the observed performance [26,31].

In this study, the Tree-structured Parzen Estimator (TPE), implemented through the Optuna framework [20], was employed. TPE separately models the distributions associated with promising and non-promising configurations and uses this information to propose new hyperparameter values. In this context, biological inspiration defines the general structure of the dynamic β trajectory, whereas Bayesian Optimization is used to identify a single global pair (m,k) intended to be applied to benchmark functions that were not involved in the tuning process.

#### 2.7.1. Holdout Protocol for Hyperparameter Tuning

To prevent the selection of the hyperparameters *m* and *k* from being influenced by the same functions subsequently used to evaluate the proposed method, the 23 benchmark functions were divided, prior to the tuning process, into two disjoint sets: a tuning set and a test set. This separation allows a single global configuration to be selected from one group of problems and its performance to be subsequently examined on functions that did not participate in the calibration process.

The benchmark functions were organized according to the classification commonly used in the evaluation of continuous metaheuristics. In particular, the original Grey Wolf Optimizer study distinguishes among unimodal, multimodal, and fixed-dimension multimodal functions, since these groups are useful for examining different search capabilities, including exploitation, exploration, and local optimum avoidance [34]. Following this classification, the benchmark suite considered in this study was organized as follows:

F1–F7:unimodalfunctions,

F8–F13:multimodalfunctions,

F14–F23:fixed-dimensionmultimodalfunctions.

The separation between tuning and test functions follows the criterion adopted in studies on the automatic configuration of continuous optimizers. Liao et al. examined the effect of tuning optimization algorithms on one benchmark set and subsequently evaluating the resulting configurations on different benchmark sets or experimental conditions [35]. This procedure makes it possible to assess whether a selected configuration maintains adequate performance outside the problems used during its calibration. A related strategy was previously used in the automatic configuration of continuous optimization algorithms, where parameter settings obtained under specific functions and dimensions were later evaluated under conditions not involved in the tuning process  [36]. Following this principle, the functions reserved for testing in the present study were not involved in the Bayesian Optimization process or in the selection of the global pair (m,k).

The partition was generated through reproducible stratified random sampling using a split seed equal to 42. Stratification ensured that the three benchmark categories were represented in both sets. Four unimodal functions, three multimodal functions, and five fixed-dimension multimodal functions were assigned to the tuning set. The remaining functions were reserved for the subsequent evaluation.

The tuning set was defined as:

$$\begin{array}{cc} F_{\mathrm{tune}}= & \{F1,F3,F6,F7,\\ & F9,F12,F13,\\ & F15,F17,F18,F22,F23\}, \end{array}$$

whereas the test set was defined as:

$$\begin{array}{cc} F_{\mathrm{test}}= & \{F2,F4,F5,\\ & F8,F10,F11,\\ & F14,F16,F19,F20,F21\}. \end{array}$$

The resulting partition is summarized in Table 3.

Therefore,

$$\left|F_{\mathrm{tune}}\right|=12,\;\left|F_{\mathrm{test}}\right|=11,$$

and the two sets satisfy:

$$F_{\mathrm{tune}}\cap F_{\mathrm{test}}=⌀,$$

$$F_{\mathrm{tune}}\cup F_{\mathrm{test}}=\left\{F1,F2,\ldots ,F23\right\}.$$

The partition was generated only once and stored before the Optuna trials were executed. Consequently, the composition of the two sets was not modified on the basis of the results observed during tuning. Functions F14–F23 retained the fixed dimensions defined for their respective problems and were not forced to use a common dimensionality.

During the tuning stage, each candidate pair $\left(m_{j},k_{j}\right)$ was evaluated exclusively on the functions belonging to $F_{\mathrm{tune}}$. The functions in $F_{\mathrm{test}}$ did not participate in the Optuna trials, in the calculation of the aggregate tuning objective, or in the selection of the hyperparameters. Once the tuning procedure was completed, a single global pair $\left(m^{*},k^{*}\right)$ was selected and frozen before the test functions were evaluated. Therefore, no function-specific values of *m* and *k* were obtained.

The same protocol was considered when defining the conditions for the baseline methods. Fully specified formulations, such as constant values of β or deterministic trajectories without tunable parameters, were applied directly. Any comparison method containing free hyperparameters must instead be configured using only $F_{\mathrm{tune}}$, without using information from the test functions. Accordingly, the results obtained on $F_{\mathrm{test}}$ represent the performance of configurations that were not specifically selected for those problems.

#### 2.7.2. Optimization Configuration

The dynamic trajectory of β is determined by the hyperparameters *m* and *k*, as defined in Equations (5) and (6). The search domains were established as:

m∈{10,11,…,30},

and

k∈[0.05,0.50].

The parameter *m* is an integer that controls the temporal location of the main transition in the arctangent curve. Owing to the endpoint normalization applied to β(t), the condition t=m does not necessarily imply β(t)=0.5. The parameter *k* is a positive real value that determines the steepness of the transition. Larger values produce a sharper decay, whereas smaller values result in a smoother and more gradual change.

Hyperparameter tuning was carried out using the Tree-structured Parzen Estimator implemented in Optuna [20]. A single global study comprising 200 trials was performed. The first 40 trials were assigned to the startup stage of the sampler, after which TPE used the accumulated observations to guide the selection of subsequent configurations. The Optuna sampler was initialized with a fixed seed of 2026. Trials were executed sequentially, with n_jobs=1, and no pruning mechanism was applied. A persistent SQLite database was used to store the study and allow its execution to be resumed without discarding completed trials.

For each trial *j*, Optuna proposed one candidate pair,

$$(m_{j},k_{j}),$$

which remained unchanged throughout all evaluations belonging to that trial. The candidate pair was evaluated only on the 12 functions included in the tuning set. Each function was executed using the following five predefined random seeds:

$$S_{\mathrm{tune}}=\left\{101,202,303,404,505\right\}.$$

Consequently, each trial involved

12tuningfunctions×5seeds=60independentLMOruns.

The same function–seed combinations were used for every candidate pair. Before each run, the pseudorandom number generators were reinitialized using a reproducible seed derived from the function identifier and the corresponding base seed. This procedure ensured that competing values of (m,k) were compared under matched initial populations and reproducible stochastic conditions. The effective seed did not depend on the trial number.

Each LMO run used a population of 50 search agents and a maximum of 100 iterations. The computational budget was defined in terms of objective-function evaluations as

$$NFE_{\max}=5050.$$

This budget included the 50 evaluations required to assess the initial population and the evaluations performed during the subsequent optimization iterations. Rather than estimating the number of evaluations solely from the population size and number of iterations, the implementation used an explicit counter that was incremented whenever the benchmark function was called. Each run was stopped when the available evaluation budget was exhausted.

**Figure 8:**
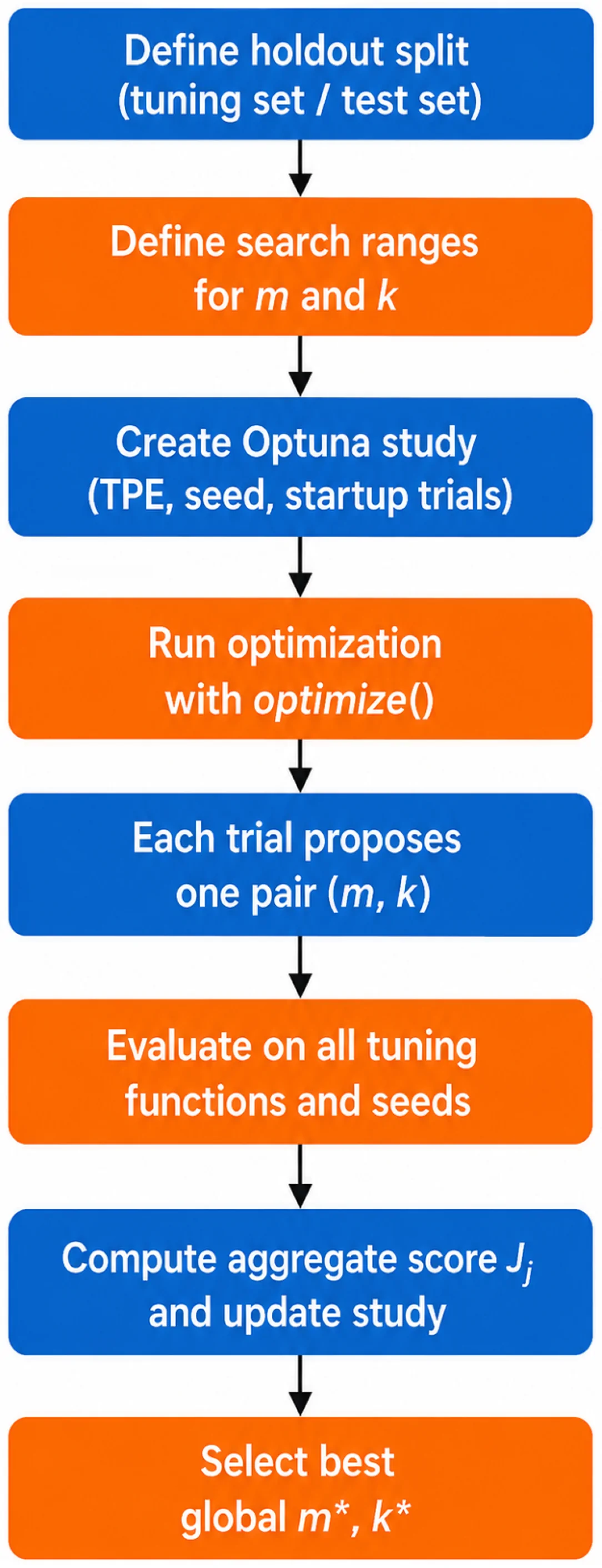
Sequential workflow of the Optuna-based procedure used to identify the best global hyperparameter pair $\left(m^{*},k^{*}\right)$.

At the end of each trial, the results obtained from the 60 runs were combined into a single aggregate objective value, denoted by $J_{j}$. This value was returned to Optuna and used to compare the candidate pair $\left(m_{j},k_{j}\right)$ with the configurations evaluated in the remaining trials. The definition of $J_{j}$ is presented in the following subsection. The overall workflow followed to identify the best global hyperparameter pair $\left(m^{*},k^{*}\right)$ is illustrated in Figure 8.

#### 2.7.3. Global Objective Function and Selection of the Best Pair

The final fitness values obtained for the different benchmark functions were not used directly as the objective of the tuning process. The benchmark functions have different search ranges, objective-value scales, and reference optima. Consequently, directly averaging their fitness values could give greater influence to functions whose objective values have a larger numerical magnitude.

To obtain a comparable measure across the benchmark functions, a global objective function was defined based on the proportion of the initial distance to the reference optimum that remained at the end of each run. This criterion follows methodological principles used to measure normalized objective reduction and to compare optimization methods through normalized performance measures [37,38]. The formulation adopted in this study was specifically adapted to the tuning of the hyperparameters *m* and *k*.

For each trial *j*, Optuna proposed a candidate pair $\left(m_{j},k_{j}\right)$. This pair was evaluated on the 12 functions belonging to the tuning set using the five random seeds defined for the procedure. Therefore, each trial involved a total of 60 LMO executions.

For a function $F_{i}$ and a seed *s*, the best fitness value found in the initial population was recorded as $f_{i,s}^{\left(0\right)}$. The best value reached at the end of the execution was denoted by

$$f_{i,s}^{\left(\mathrm{final}\right)}\left(m_{j},k_{j}\right).$$

Let $f_{i}^{\mathrm{ref}}$ denote the reference optimum registered for function $F_{i}$. The initial distance from this value was calculated as

$$d_{i,s}^{\left(0\right)}=\left|f_{i,s}^{\left(0\right)}-f_{i}^{\mathrm{ref}}\right|,$$

whereas the distance remaining at the end of the execution was calculated as

$$d_{i,s}^{\left(\mathrm{final}\right)}\left(m_{j},k_{j}\right)=\left|f_{i,s}^{\left(\mathrm{final}\right)}\left(m_{j},k_{j}\right)-f_{i}^{\mathrm{ref}}\right|.$$

Using these quantities, the normalized remaining optimality gap at the end of the execution was defined as

$$r_{i,s}\left(m_{j},k_{j}\right)=\frac{d_{i,s}^{\left(\mathrm{final}\right)}\left(m_{j},k_{j}\right)}{\max\left(d_{i,s}^{\left(0\right)},\varepsilon \right)},\;\varepsilon =10^{-12}.$$

The term ε prevents division by zero when the best individual in the initial population coincides with the reference value. A value of $r_{i,s}$ close to zero indicates that the execution ended near the reference optimum. Higher values indicate that a larger proportion of the initial distance remained after the search. The resulting value was not clipped or subjected to any additional transformation.

The global objective was calculated in two stages. First, for each function $F_{i}$, the normalized values obtained from the five seeds were averaged:

$$R_{i}\left(m_{j},k_{j}\right)=\frac{1}{\left|S_{\mathrm{tune}}\right|}\sum_{s\in S_{\mathrm{tune}}}r_{i,s}\left(m_{j},k_{j}\right),$$

where

$$\left|S_{\mathrm{tune}}\right|=5.$$

The results obtained for the 12 tuning functions were then combined to calculate the global objective value associated with trial *j*:

$$J_{j}=\frac{1}{\left|F_{\mathrm{tune}}\right|}\sum_{F_{i}\in F_{\mathrm{tune}}}R_{i}\left(m_{j},k_{j}\right),$$

with

$$\left|F_{\mathrm{tune}}\right|=12.$$

Because all functions were evaluated using the same number of seeds, the same expression can also be written as

$$J_{j}=\frac{1}{60}\sum_{F_{i}\in F_{\mathrm{tune}}}\sum_{s\in S_{\mathrm{tune}}}r_{i,s}\left(m_{j},k_{j}\right).$$

However, the implementation first calculated the mean across the five seeds for each function and then calculated the mean across the 12 functions. In this way, each tuning function contributed equally to the global objective value $J_{j}$.

The value $J_{j}$ was returned to Optuna at the end of each trial. Since the study was configured as a minimization problem, candidate pairs producing lower values of $J_{j}$ were considered more favorable. Once all trials had been processed, the completed trial with the lowest global objective value was identified as

$$j^{*}=\underset{j\in T_{\mathrm{complete}}}{\arg\min}\;J_{j}.$$

The best global pair was then defined as

$$\left(m^{*},k^{*}\right)=\left(m_{j^{*}},k_{j^{*}}\right),$$

and its corresponding objective value was defined as

$$J^{*}=J_{j^{*}}.$$

The pair $\left(m^{*},k^{*}\right)$ was selected only once for the complete set of functions. Therefore, no function-specific values of *m* and *k* were obtained. The functions belonging to the test set were not involved in the calculation of $r_{i,s}$, $R_{i}$, or $J_{j}$, and they did not participate in the selection of the best pair. After the tuning stage was completed, the selected values were kept fixed for their subsequent evaluation on the test set.

### 2.8. Hyperparameter Estimation for β

After completion of the Bayesian Optimization procedure, trial 196 produced the lowest global objective value among the evaluated configurations. The corresponding hyperparameter pair was

(7) $$\left(m^{*},k^{*}\right)=\left(10,\;0.08479497153015933\right),$$

with an objective value of

(8) $$J^{*}=0.00830912399344953.$$

This configuration was therefore selected as the global hyperparameter setting within the predefined search domain. The pair $\left(m^{*},k^{*}\right)$ was subsequently frozen and used without further tuning for the evaluation on the held-out test functions.

The value $J^{*}$ corresponds to the mean of the normalized results $R_{i}$ obtained for the 12 functions in the tuning set. For each function, $R_{i}$ represents the mean normalized remaining optimality gap across the five seeds used in the experiment. Therefore, a lower value of $R_{i}$ indicates that the final fitness was closer to the known optimum relative to the distance observed in the initial population. To facilitate the interpretation of these results, the relative reduction in the distance to the known optimum was calculated as

$${\mathrm{Reduction}}_{i}=\left(1-R_{i}\right)\times 100.$$

For example, for F3,

$${\mathrm{Reduction}}_{F3}=\left(1-0.02101148398666236\right)\times 100=97.898851601\%.$$

Similarly, for F9,

$${\mathrm{Reduction}}_{F9}=\left(1-0.07214391738664105\right)\times 100=92.785608261\%.$$

Table 4 presents the $R_{i}$ values obtained with the selected configuration and their corresponding reduction percentages. The $R_{i}$ values are reported in scientific notation with six digits after the decimal point in the mantissa. The percentages were calculated using the values stored at full numerical precision.

The $R_{i}$ values show that the selected configuration substantially reduced the initial distance to the known reference optimum for the functions used during tuning. Ten of the twelve functions achieved a reduction equal to or greater than 99%. The exceptions were F3 and F9, with approximate reductions of 97.90% and 92.79%, respectively. Thus, although the proportion of the remaining distance varied across functions, it remained below 7.22% in all cases. At the global level, the value

$$J^{*}=0.0083091240$$

indicates that, on average, approximately 0.8309% of the initial distance to the known reference optimum remained at the end of the search. The corresponding average reduction was

$$1-J^{*}=0.9916908760,$$

which is equivalent to approximately 99.17%. This percentage represents the average reduction in the initial distance and should not be interpreted as a direct percentage of the fitness value.

#### Computational Cost of the Tuning Process

The computational cost was determined from the number of trials, the benchmark functions included in the tuning set, the number of seeds, and the evaluation budget assigned to each run. Each candidate pair (m,k) was evaluated on 12 functions using five seeds. Therefore, each trial comprised

12×5=60

LMO runs. Since the study considered 200 trials, the total number of runs was

200×60=12,000.

Each run was assigned a budget of 5050 objective-function evaluations. Consequently, the cost of each trial was

60×5050=303,000

evaluations, whereas the total cost of the tuning process was

200×12×5×5050=60,600,000

objective-function evaluations. All 200 trials were completed without failures; therefore, the actual number of evaluations was equal to the calculated value. The recorded wall-clock time was 11,434.05 s, equivalent to approximately 3.18 h.

### 2.9. Comparative Configurations for β

The previous sections introduced a bio-inspired formulation for the parameter β and determined its hyperparameters through Bayesian Optimization. However, to analyze the behavior of the proposed approach, it is necessary to define the alternative β configurations against which it will be compared during the experimental stage. Since the original LMO algorithm does not provide a formal definition of β, a direct comparison with the original formulation is not possible. Therefore, the experimental analysis considers different strategies for defining β within the LMO framework.

For this purpose, six strategy families for defining β within the LMO algorithm are considered, resulting in nine evaluated configurations:

(i) A constant β with values of 0.1, 0.25, 0.5, and 1. These configurations are referred to in the experiments as LMOB01, LMOB025, LMOB05, and LMOB1, respectively.

(ii) A nonlinear decreasing β schedule adapted from the inertia-weight control strategy employed in modified PSO variants [39,40]. In the original formulation, this schedule regulates the PSO inertia weight *w*. In the present study, its temporal structure was transferred to the parameter β of LMO to provide a non-bio-inspired dynamic baseline.

(9) $$\beta_{d}\left(t\right)=\beta_{\min}+\left(\beta_{\max}-\beta_{\min}\right){\left(1-\frac{t}{{\mathrm{Iter}}_{\max}}\right)}^{2}.$$

where $\beta_{\min}=0$ and $\beta_{\max}=1$.

This configuration is referred to as LMOQUADRATICDECAY in the experiments.

(iii) A linear decreasing β schedule derived from the convergence-control mechanism of the Grey Wolf Optimizer (GWO) [34]. In GWO, the coefficient vector A is defined as

(10) $$A\left(t\right)=2a\left(t\right)r_{1}-a\left(t\right),$$

where $r_{1}$ is a random vector with components in [0,1], and a is linearly decreased from 2 to 0 over the course of the iterations. The magnitude of A regulates the search behavior in GWO: values satisfying |A|>1 promote divergence and exploration, whereas |A|<1 promote convergence and exploitation.

For $t\in [0,{\mathrm{Iter}}_{\max}]$, the linear decrease described in GWO can be expressed as

(11) $$a\left(t\right)=2\left(1-\frac{t}{{\mathrm{Iter}}_{\max}}\right).$$

To transfer only this deterministic temporal schedule to LMO, without incorporating the stochastic movement operator of GWO, a(t) is normalized to the interval [0,1] and used to define β as

(12) $$\beta_{\mathrm{GWO}}\left(t\right)=\frac{a(t)}{2}=1-\frac{t}{{\mathrm{Iter}}_{\max}}.$$

This configuration is referred to as LMOGWO in the experiments.

(iv) A normalized exponentially decreasing β schedule was included as a nonlinear monotonic baseline. This configuration is referred to as LMOEXP. Unlike the proposed bio-inspired formulation, this strategy does not contain adjustable parameters and depends only on the normalized iteration progress. It is defined as

(13) $$\beta_{\mathrm{EXP}}\left(t\right)=\frac{\exp\left(1-\frac{t}{{\mathrm{Iter}}_{\max}}\right)-1}{\exp(1)-1},$$

where *t* is the current iteration and ${\mathrm{Iter}}_{\max}$ is the maximum number of iterations. The normalization guarantees that $\beta_{\mathrm{EXP}}\left(0\right)=1$ and $\beta_{\mathrm{EXP}}\left({\mathrm{Iter}}_{\max}\right)=0$. This schedule produces a faster reduction during the initial part of the optimization process, followed by a progressively smoother decrease toward zero.

(v) An adaptive control mechanism inspired by AIWPSO was incorporated to determine β from the search performance observed during each iteration [41]. The value β(t) is used in the LMO movement equation to generate the candidate solutions.

For a minimization problem, the success indicator of candidate $y_{i}\left(t\right)$ is defined as

(14) $$s_{i}\left(t\right)=\left\{\begin{array}{cc} 1, & f\left(y_{i}\left(t\right)\right)<f\left(x_{w}\left(t\right)\right),\\ 0, & \mathrm{otherwise}, \end{array}\right.$$

where $x_{w}\left(t\right)$ denotes the current worst individual. The success rate of the population is then computed as

(15) $$P_{s}\left(t\right)=\frac{1}{N}\sum_{i=1}^{N}s_{i}\left(t\right),$$

where *N* is the population size. The value of β for the next iteration is obtained from

(16) $$\beta \left(t+1\right)=\beta_{\min}+\left(\beta_{\max}-\beta_{\min}\right)P_{s}\left(t\right).$$

where $\beta_{\min}=0$ and $\beta_{\max}=1$.

Successful candidates replace the current worst individual, whereas unsuccessful candidates are discarded. Therefore, β is updated according to the proportion of candidate solutions accepted during the preceding iteration. This configuration is referred to as LMOAIWPSO in the experiments.

(vi) The bio-inspired dynamic β proposed in this work, based on the normalized arctangent formulation described in Section 2.6. The trajectory is parameterized by the globally tuned hyperparameters $m^{*}=10$ and $k^{*}=0.0847949715$, reported in Equation (7) A single global pair $\left(m^{*},k^{*}\right)$ is used for all test functions, with no function-specific tuning. This configuration is referred to as LMOBIOINSPIRED in the experiments.

Overall, these six strategy families result in nine LMO variants, which constitute the comparative framework used in the experimental evaluation presented in Section 3.

## 3. Results

### 3.1. Experimental Setup and Computational Time

The final evaluation was conducted on the 11 functions reserved for the testing stage: F2, F4, F5, F8, F10, F11, F14, F16, F19, F20, and F21. Nine LMO variants were compared: LMOBIOINSPIRED, LMOB1, LMOB05, LMOB025, LMOB01, LMOQUADRATICDECAY, LMOGWO, LMOEXP, and LMOAIWPSO.

Each variant was independently executed 31 times on each test function, using a population of 50 individuals and a maximum of 100 iterations. Functions F2, F4, F5, F8, F10, and F11 were evaluated with D=30. For the fixed-dimension functions, D=2 was used for F14 and F16, D=3 for F19, D=6 for F20, and D=4 for F21. Overall, the experiment comprised 3069 independent executions.

All LMO variants were implemented in Python 3.11.7 and executed on a computer equipped with an Intel Core i9-10900K processor (Intel Corporation, Santa Clara, CA, USA) running at 3.70 GHz, 64 GB of RAM, and the Windows 10 operating system. The cumulative execution time across all 3069 runs was 3820.167 s, equivalent to approximately 1 h, 3 min, and 40 s.

### 3.2. Fitness Results

Table 5 presents the first-ranked variant for each test function according to the median final fitness obtained from the 31 independent runs. The rankings were determined using the full-precision fitness values stored in the experimental database, whereas the median values reported in the table are rounded to four significant digits for presentation. LMOBIOINSPIRED achieved the lowest median fitness as the unique first-ranked variant on F2, F4, F5, F10, F11, F19, F20, and F21. LMOB01 was the unique first-ranked variant on F8, whereas LMOQUADRATICDECAY achieved the lowest median fitness on F14 and F16. Therefore, LMOBIOINSPIRED reached the best median performance on 8 of the 11 test functions. These descriptive results are complemented by the statistical analysis presented in the following subsection.

**Figure 9:**
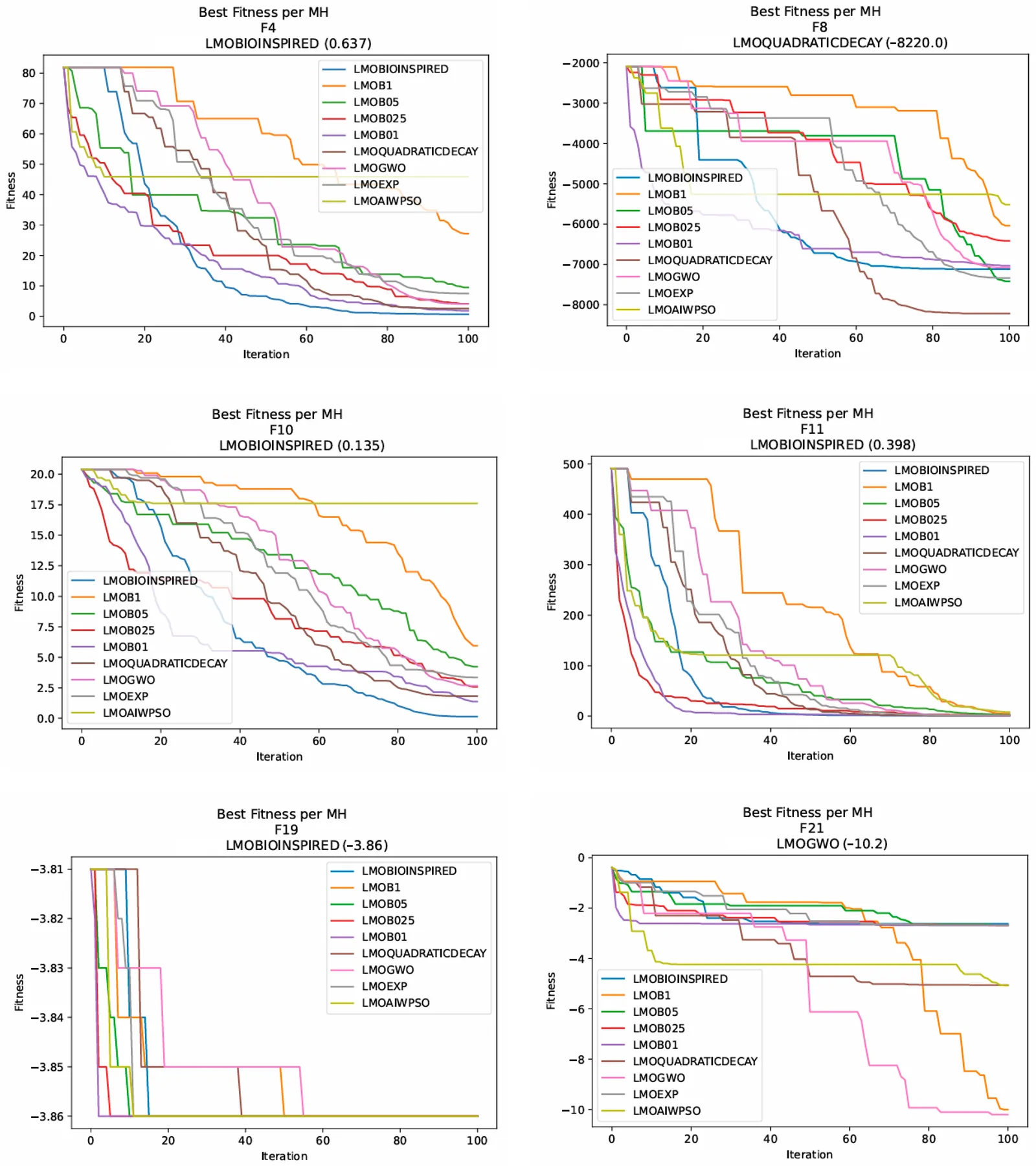
Best-fitness trajectories from the last processed run for the nine LMO variants on functions F4, F8, F10, F11, F19, and F21.

Figure 9 presents, for illustrative purposes, the best-so-far fitness trajectories of the nine variants on functions F4, F8, F10, F11, F19, and F21. For each function–variant combination shown, the plotted trajectory corresponds to the last processed run among the 31 independent executions performed on the corresponding benchmark function. These trajectories are included to illustrate the convergence behavior of the evaluated configurations and are not used as the basis for the statistical comparison. The statistical analysis presented in Section 3.3 is based on the median final fitness obtained from the 31 independent runs for each function–variant combination.

Considering the last processed runs shown in Figure 9, LMOBIOINSPIRED achieved the lowest final best-so-far fitness on F4, with a value of approximately 0.6372. On F8, the lowest final value among the displayed trajectories was obtained by LMOQUADRATICDECAY, which reached approximately −8221, whereas LMOGWO obtained the lowest final value on F21, with approximately −10.15. LMOBIOINSPIRED achieved the lowest final values on F10, F11, and F19, with values of approximately 0.1348, 0.3981, and −3.863, respectively. The corresponding trajectories show a progressive reduction in fitness followed by stabilization during the final iterations. These values correspond only to the illustrative last processed runs shown in the figure and should not be interpreted as the median performance across the 31 independent executions.

### 3.3. Statistical Results for the Test Functions

The statistical analysis was conducted on the 11 functions reserved for the testing stage: F2, F4, F5, F8, F10, F11, F14, F16, F19, F20, and F21. For each function–variant combination, the median final fitness obtained from 31 independent runs was used as the representative performance measure. The test functions were treated as blocks, while the nine LMO variants were considered the methods under comparison.

#### 3.3.1. Global Friedman Test

The non-parametric Friedman test was applied to determine whether the evaluated variants exhibited equivalent performance across the test functions. The Friedman statistic is defined as

(17) $$\chi_{F}^{2}=\frac{12N}{k(k+1)}\sum_{j=1}^{k}R_{j}^{2}-3N\left(k+1\right),$$

where *N* denotes the number of test functions, *k* is the number of variants, and $R_{j}$ is the average rank of variant *j*. In the present analysis, N=11 and k=9.

A correction for tied ranks was considered. The correction factor is given by

(18) $$C=1-\frac{\sum_{i=1}^{N}\sum_{g}\left(t_{ig}^{3}-t_{ig}\right)}{N(k^{3}-k)},$$

where $t_{ig}$ is the number of variants belonging to tie group *g* for function *i*. The corrected Friedman statistic is then obtained as

(19) $$\chi_{F,c}^{2}=\frac{\chi_{F}^{2}}{C}.$$

When the median fitness values stored at full numerical precision were used, no identical median values were observed among the variants within any of the test functions. Consequently, the tie-correction factor was C=1, and the corrected statistic was identical to the uncorrected Friedman statistic. For the test data, the resulting statistic was

(20) $$\chi_{F,c}^{2}=60.4364,\;df=8,\;p=3.8269\times 10^{-10}.$$

Because the *p*-value was below the significance level α=0.05, the null hypothesis of equivalent performance was rejected. Thus, the Friedman test identified statistically significant differences among the nine variants over the test functions.

The magnitude of the global differences was assessed using Kendall’s coefficient of concordance:

(21) $$W=\frac{\chi_{F,c}^{2}}{N(k-1)}.$$

Substituting the values obtained in the Friedman test gives

(22) $$W=\frac{60.4364}{11(9-1)}=0.6868.$$

Kendall’s *W* ranges from 0 to 1, with larger values indicating greater concordance in the ranking of the methods across the test functions. The obtained value, W=0.6868, indicates a relatively high degree of concordance among the rankings of the evaluated variants across the test functions.

#### 3.3.2. Average Ranks

For each test function, the variants were ranked according to their median final fitness. Rank 1 was assigned to the lowest fitness value and therefore to the best-performing variant. When two or more variants obtain the same median fitness, the corresponding ranks are averaged. The average rank of variant *j* was calculated as

(23) $$R_{j}=\frac{1}{N}\sum_{i=1}^{N}R_{ij},$$

where $R_{ij}$ denotes the rank assigned to variant *j* on function *i*, and N=11 is the number of test functions.

Table 6 presents the average ranks obtained by each variant across the 11 held-out test functions. LMOBIOINSPIRED achieved the lowest average rank, with a value of 1.7273. The next positions were occupied by LMOQUADRATICDECAY, LMOEXP, and LMOGWO, with average ranks of 2.0909, 3.8182, and 4.4545, respectively. These results indicate that LMOBIOINSPIRED exhibited the most favorable overall ranking across the 11 held-out test functions, followed by LMOQUADRATICDECAY.

The average ranks provide an overall ordering of the variants across the test functions. However, they do not determine which pairwise differences are statistically significant. Therefore, a post-hoc analysis was subsequently performed.

#### 3.3.3. Post-Hoc Wilcoxon Test with Holm Correction

Following the significant Friedman result, pairwise comparisons were conducted between LMOBIOINSPIRED and each of the remaining eight variants. Two-sided Wilcoxon signed-rank tests were applied to the median fitness values obtained for the 11 test functions.

For the comparison between LMOBIOINSPIRED, denoted by *B*, and another variant *j*, the difference for function *i* was defined as

(24) $$d_{i}=f_{i,j}^{e}-f_{i,B}^{e},$$

where $f_{i,j}^{e}$ denotes the median final fitness of variant *j* on function *i*, and $f_{i,B}^{e}$ is the corresponding median for LMOBIOINSPIRED. Under this definition, a positive value of $d_{i}$ indicates a lower fitness value for LMOBIOINSPIRED.

Differences equal to zero were treated as ties and excluded from the signed-rank calculation. For the remaining differences, ranks $\rho_{i}$ were assigned according to their absolute values. The positive and negative rank sums were calculated as

(25) $$W^{+}=\sum_{d_{i}>0}\rho_{i},\;W^{-}=\sum_{d_{i}<0}\rho_{i}.$$

The Wilcoxon statistic was defined as

(26) $$T=\min\left(W^{+},W^{-}\right).$$

Exact two-sided *p*-values were calculated from the distribution of the non-zero signed ranks. Since m=8 pairwise comparisons were performed, the resulting *p*-values were adjusted using Holm’s step-down procedure. After arranging the original values as

(27) $$p_{\left(1\right)}\leq p_{\left(2\right)}\leq \cdots \leq p_{\left(m\right)},$$

the adjusted value at position *i* was calculated as

(28) $$p_{\mathrm{Holm},(i)}=\max_{\ell \leq i}\left[\min\left(1,\left(m-\ell +1\right)p_{\left(\ell \right)}\right)\right].$$

The magnitude of each pairwise difference was quantified using the rank-biserial correlation:

(29) $$r_{rb}=\frac{W^{+}-W^{-}}{W^{+}+W^{-}}.$$

According to the definition adopted for $d_{i}$, positive values of $r_{rb}$ favor LMOBIOINSPIRED. A value of $r_{rb}=1$ indicates that all functions with non-zero differences favored this variant.

In Table 7, W/T/L denotes the number of wins, ties, and losses obtained by LMOBIOINSPIRED, respectively. No ties were observed in the pairwise comparisons based on the full-precision median fitness values.

After applying Holm’s correction, statistically significant differences were found against LMOB025 and LMOAIWPSO. The comparison with LMOB025 yielded the lowest adjusted value, with $p_{\mathrm{Holm}}=0.00781$. LMOBIOINSPIRED recorded eleven wins and no losses in this comparison, with a rank-biserial correlation of $r_{rb}=1.0000$, indicating that all 11 test functions favored LMOBIOINSPIRED.

The comparison with LMOAIWPSO was also statistically significant, with $p_{\mathrm{Holm}}=0.01367$. LMOBIOINSPIRED obtained ten wins and one loss, with $r_{rb}=0.9697$, indicating a strong predominance of positive signed ranks in favor of LMOBIOINSPIRED.

No statistically significant differences were detected after Holm’s correction against LMOQUADRATICDECAY, LMOEXP, LMOGWO, LMOB01, LMOB05, or LMOB1. Although the unadjusted *p*-values were below 0.05 in all six comparisons, the Holm-adjusted value was $p_{\mathrm{Holm}}=0.05859$, which is above the significance level α=0.05. LMOBIOINSPIRED nevertheless obtained nine wins and two losses against LMOQUADRATICDECAY, and ten wins and one loss against each of the other five variants. These results indicate a favorable descriptive tendency for LMOBIOINSPIRED, but the corresponding pairwise differences did not remain statistically significant after correction for multiple comparisons.

### 3.4. Ablation Study

To isolate the effect of the temporal profile of β, LMOBIOINSPIRED was compared with three reduced configurations. LMOSTEP replaces the gradual transition with an abrupt change from β=1 to β=0 after $m^{*}=10$; LMOGWO applies the linear schedule $\beta \left(t\right)=1-t/{\mathrm{Iter}}_{\max}$; and LMOB01 maintains β=0.1 throughout the search. All remaining components of LMO were kept unchanged. The 2852 scheduled executions were completed, corresponding to 23 functions, four variants, and 31 independent runs, with a population of 50 individuals and 100 iterations. Since 12 functions had previously been used to estimate $m^{*}=10$ and $k^{*}=0.08479497153015933$, the inferential analysis was restricted to the 11 held-out test functions.

Table 8 presents the median final fitness and average rank obtained by each configuration over 31 runs on the 11 held-out test functions.

LMOBIOINSPIRED achieved the best average rank. The tie-corrected Friedman test identified significant global differences among the four configurations ($\chi_{F,c}^{2}=18.3176$, df=3, $p=3.7824\times 10^{-4}$), with Kendall’s coefficient of concordance W=0.5551.

Table 9 reports the pairwise Wilcoxon signed-rank comparisons between LMOBIOINSPIRED and each ablated configuration. After Holm’s correction, only the difference against LMOSTEP remained statistically significant. The mean exploration/exploitation percentages were 17.47/82.53% for LMOBIOINSPIRED, 10.29/89.71% for LMOSTEP, 32.42/67.58% for LMOGWO, and 6.72/93.28% for LMOB01. The corresponding mean execution times were comparable at 2.021, 2.022, 2.062, and 2.078 s.

Overall, the arctangent trajectory significantly outperformed the abrupt transition and achieved the best overall rank. However, its differences from the linear schedule and the low constant value did not remain statistically significant after correction for multiple comparisons.

## 4. Results on the CEC 2022 Benchmark Suite

### 4.1. Experimental Setup and Computational Time

The evaluation on CEC 2022 was conducted using the 12 benchmark functions, F1–F12. The same nine LMO variants considered in the previous evaluation were compared: LMOBIOINSPIRED, LMOB1, LMOB05, LMOB025, LMOB01, LMOQUADRATICDECAY, LMOGWO, LMOEXP, and LMOAIWPSO.

Each variant was independently executed 31 times on each function, using a population of 50 individuals and a maximum of 100 iterations. All CEC 2022 functions were evaluated with D=10 and search bounds of [−100,100]. Overall, the experiment comprised 3348 independent executions.

For LMOBIOINSPIRED, the hyperparameter configuration obtained during the previous tuning stage was retained without modification, with $m^{*}=10$ and $k^{*}=0.08479497153015933$. No additional tuning was performed on the CEC 2022 functions, so this benchmark suite was used as an additional evaluation of the previously selected configuration.

The cumulative execution time across all 3348 runs was 56,489.906 s, equivalent to approximately 15 h, 41 min, and 30 s.

### 4.2. Fitness Results

**Figure 10:**
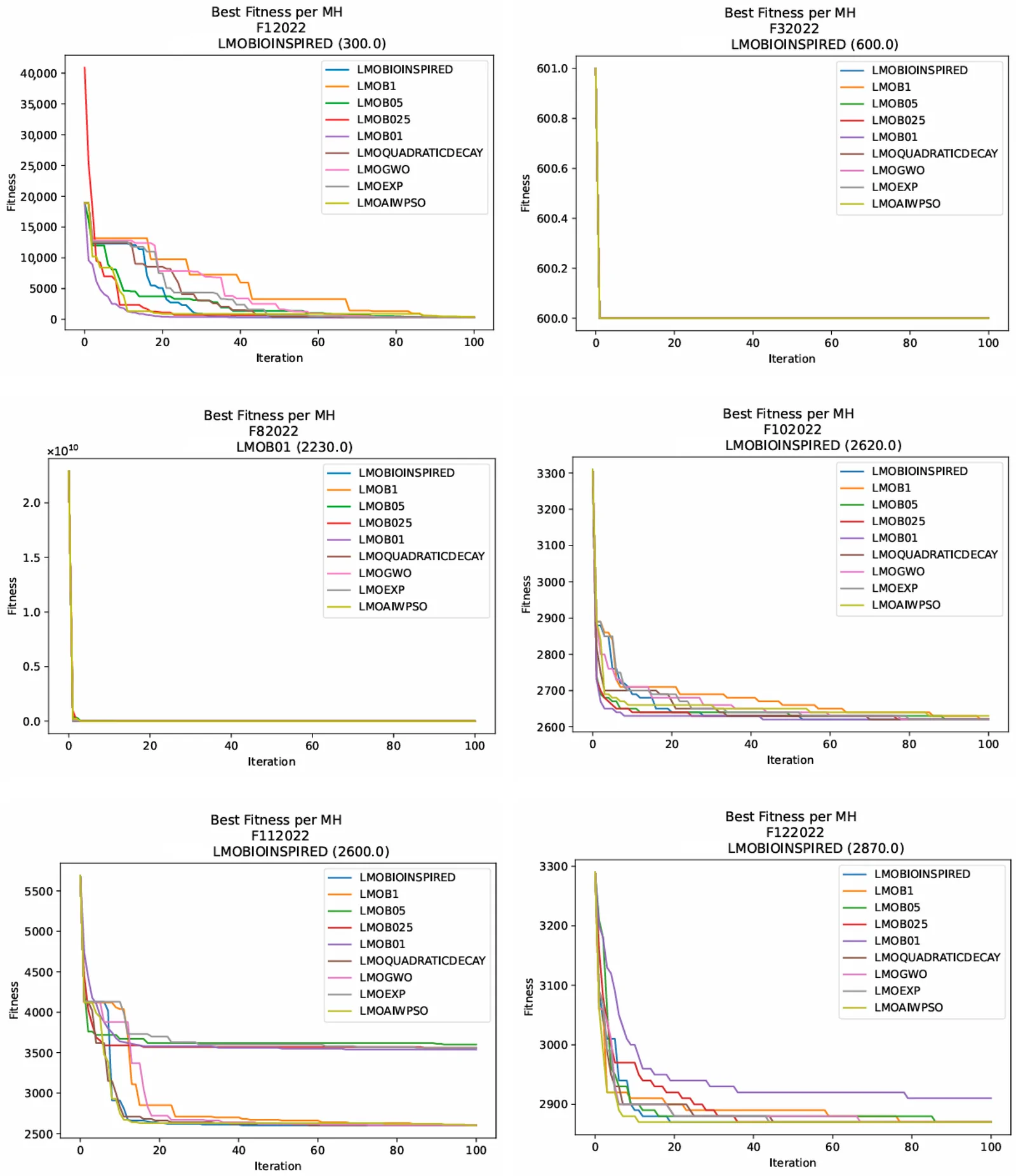
Best-fitness trajectories from the last processed run for the nine LMO variants on functions F1, F3, F8, F10, F11, and F12.

Figure 10 presents, for illustrative purposes, the best-so-far fitness trajectories of the nine variants on functions F1, F3, F8, F10, F11, and F12 from the CEC 2022 benchmark suite. For each function–variant combination shown, the plotted trajectory corresponds to the last processed run among the 31 independent executions performed on the corresponding benchmark function. These trajectories are included to illustrate the convergence behavior of the evaluated configurations and are not used as the basis for the statistical comparison. The statistical analysis presented in the following subsections is based on the median final fitness obtained from the 31 independent runs for each function variant combination.

Considering the last processed runs shown in Figure 10, LMOBIOINSPIRED achieved the lowest final best-so-far fitness on F1, F3, and F11, with values of approximately 300.0, 600.0, and 2600, respectively. On F8, the lowest final value among the displayed trajectories was obtained by LMOB01, which reached approximately 2227. On F10, the best final trajectory among the illustrated runs was obtained by LMOB025, with a value of approximately 2621, whereas on F12 the lowest final value was obtained by LMOEXP, with approximately 2865. Taken together, the trajectories show a progressive reduction in fitness followed, in most cases, by stabilization during the final iterations. These values correspond only to the illustrative last processed runs shown in the figure and should not be interpreted as the median performance across the 31 independent executions.

Table 10 presents the first-ranked variant for each test function according to the median final fitness obtained from the 31 independent runs. The rankings were determined using the full-precision fitness values stored in the experimental database, whereas the median values reported in the table are rounded to four significant digits for presentation. LMOBIOINSPIRED achieved the lowest median fitness on F1, F3, F5, and F11, whereas LMOB01 was the first-ranked variant on F2, F6, F8, and F10. LMOGWO achieved the lowest median fitness on F4, LMOQUADRATICDECAY on F7, LMOB025 on F9, and LMOEXP on F12. Therefore, LMOBIOINSPIRED and LMOB01 shared the strongest descriptive performance, each reaching the first position on four of the 12 test functions. These descriptive results are complemented by the statistical analysis presented in the following subsection.

### 4.3. Global Friedman Test

To determine whether the nine variants exhibited equivalent performance on the CEC 2022 benchmark suite, the non-parametric Friedman test was applied to the median final fitness values obtained for the 12 functions. The test yielded a statistic of 44.6444, with eight degrees of freedom and $p=4.2973\times 10^{-7}$. Therefore, the null hypothesis was rejected at the 0.05 significance level, confirming statistically significant global differences among the evaluated β-control strategies. Kendall’s coefficient of concordance was W=0.4650, indicating a meaningful, although not complete, degree of agreement in the ranking of the variants across the evaluated functions. This global result does not imply that every configuration differs significantly from all the others. Consequently, the analysis was complemented by the average ranks and the post-hoc comparisons reported in the following subsections.

### 4.4. Average Ranks

Following the same ranking procedure described previously for the test functions, the nine variants were ranked on each CEC 2022 function according to the median final fitness obtained from the 31 independent runs. Rank 1 was assigned to the lowest median fitness, and the ranks obtained across the 12 functions were subsequently averaged for each variant.

In Table 11, LMOBIOINSPIRED achieved the lowest average rank, with a value of 3.0833. The next positions were occupied by LMOQUADRATICDECAY and LMOEXP, with average ranks of 3.5000 and 3.5833, respectively. LMOB025, LMOB01, and LMOGWO followed with average ranks of 4.1667, 4.2500, and 4.4167. LMOB1 and LMOAIWPSO obtained the same average rank of 7.9167.

These results indicate that LMOBIOINSPIRED exhibited the most favorable overall ranking across the 12 CEC 2022 functions. Nevertheless, the average ranks provide an overall ordering of the variants and do not establish which pairwise differences are statistically significant. This issue is examined through the post-hoc analysis presented in the following subsection.

### 4.5. Post-Hoc Wilcoxon Test with Holm Correction

Following the significant Friedman result, pairwise comparisons were conducted between LMOBIOINSPIRED and each of the remaining eight variants. The same two-sided Wilcoxon signed-rank procedure and Holm correction described previously for the test functions were applied. The comparisons were based on the median final fitness values obtained for the 12 CEC 2022 functions, using the full-precision values stored in the experimental database.

In Table 12, W/T/L denotes the number of wins, ties, and losses obtained by LMOBIOINSPIRED, respectively. No ties were observed in any of the pairwise comparisons based on the full-precision median fitness values.

After applying Holm’s correction, a statistically significant difference was found only in the comparison with LMOAIWPSO. LMOBIOINSPIRED achieved 12 wins and no losses in this comparison, with p=0.00049, $p_{\mathrm{Holm}}=0.00391$, and a rank-biserial correlation of $r_{rb}=1.0000$. Thus, all 12 CEC 2022 functions favored LMOBIOINSPIRED in this comparison.

Against LMOB1, LMOBIOINSPIRED obtained 11 wins and one loss, with p=0.00928 and $r_{rb}=0.8205$. However, after the Holm adjustment, the corrected value increased to $p_{\mathrm{Holm}}=0.06494$, and the difference therefore did not remain statistically significant at α=0.05.

No statistically significant differences were detected after correction against LMOQUADRATICDECAY, LMOEXP, LMOGWO, LMOB01, LMOB025, or LMOB05. LMOBIOINSPIRED nevertheless obtained ten wins and two losses against LMOB05, nine wins and three losses against LMOEXP, eight wins and four losses against both LMOQUADRATICDECAY and LMOGWO, seven wins and five losses against LMOB025, and six wins and six losses against LMOB01. These results show a favorable descriptive tendency for LMOBIOINSPIRED in several comparisons; however, after controlling for multiple comparisons, statistical significance was retained only against LMOAIWPSO.

## 5. Implementing Bio-Inspired β in GWO

To examine whether the proposed control mechanism could be transferred to another metaheuristic, the bio-inspired β trajectory was incorporated into the control parameter *a* of GWO without modifying its update operators. The hyperparameters *m* and *k* were tuned using Optuna with a TPE sampler. The procedure comprised 200 trials, including 40 initial trials, with m∈{10,…,30} and k∈[0.05,0.50]. Each configuration was evaluated using a population of 50 individuals, 100 iterations, and the five fixed seeds 101, 202, 303, 404, and 505.

A holdout protocol was used to separate tuning from evaluation. Functions F1, F3, F6, F7, F9, F12, F13, F15, F17, F18, F22, and F23 were used for tuning. Functions F2, F4, F5, F8, F10, F11, F14, F16, F19, F20, and F21 were reserved for the subsequent evaluation and did not participate in hyperparameter selection. Since each trial involved 12 functions and five seeds, 60 runs were performed per configuration, resulting in 12,000 runs and 60,600,000 objective-function evaluations. All 200 trials were completed successfully. The wall-clock time was 3695.46 s, or approximately 1.03 h.

The best configuration was obtained in trial 155, with $m^{*}=29$, $k^{*}=0.05719374814281837$, and $J^{*}=0.0043031709425245274$. The value of *m*, which is close to the upper bound of the evaluated interval, places the regime change toward the end of the permitted window for this parameter. The comparatively low value of *k* describes a gradual transition rather than an abrupt decay. The reported value of *J* was the lowest mean normalized remaining optimality gap found during tuning.

The selected configuration was subsequently compared with the original GWO on the 11 held-out functions. Both algorithms were independently executed 31 times per function, using a population of 50 individuals and 100 iterations. All 682 scheduled runs were completed without failures, with a cumulative execution time of 239.41 s. Table 13 reports the final median fitness and the corresponding ranks. Lower fitness and rank values indicate better performance.

GWOBIOINSPIRED achieved the lowest median fitness on F8, F14, and F21, whereas GWO ranked first on F2, F4, F5, F10, and F11. On F16, F19, and F20, the two methods produced the same median at the precision available in the exported fitness files. The resulting average ranks were 1.4091 for GWO and 1.5909 for GWOBIOINSPIRED.

After accounting for tied ranks, the corrected Friedman test yielded $\chi_{F,c}^{2}=0.5000$, with df=1 and p=0.4795. Thus, the null hypothesis of equivalent performance was not rejected. Kendall’s coefficient of concordance was W=0.0455, indicating a small overall effect. Since only two methods were compared, a multiple-comparison post-hoc procedure was not strictly required. Nevertheless, to maintain consistency with the preceding analysis, an exact two-sided Wilcoxon signed-rank test was applied to the per-function medians, excluding the three zero differences. The test produced T=15.0, p=0.7422, and a rank-biserial correlation of $r_{\mathrm{rb}}=-0.1667$. Because there was only one pairwise comparison, Holm’s adjustment did not change the result, giving $p_{\mathrm{Holm}}=0.7422$. The win/tie/loss record of GWOBIOINSPIRED against GWO was 3/3/5.

These findings show that the bio-inspired control schedule can be incorporated into GWO without altering its general structure and can improve performance on some functions. However, the advantage was neither uniform nor statistically significant relative to the original linear schedule. The experiment therefore supports the transferability of the control mechanism, but it does not establish an overall superiority of GWOBIOINSPIRED.

## 6. Conclusions and Future Work

This study examined a complementary use of bio-inspiration: employing biological phenomena to model the temporal evolution of internal parameters that influence exploration and exploitation dynamics, rather than using them exclusively to construct new algorithms or search operators. Using LMO as a case study, the progressive transition observed during bird landing was transferred to a normalized trajectory for β, whose hyperparameters were determined through Bayesian optimization.

The initial evaluation considered 23 benchmark functions, of which 12 were used for tuning and 11 were reserved for independent testing. On the held-out functions, LMOBIOINSPIRED achieved the best average rank (1.7273), while the Friedman test identified significant global differences among the nine evaluated configurations ($\chi_{F,c}^{2}=60.4364$, $p=3.8269\times 10^{-10}$). After Holm’s correction, significant differences were found against two of the eight alternatives, LMOB025 and LMOAIWPSO. Although not all pairwise comparisons reached statistical significance, the results show a favorable tendency and support parameter bio-inspiration as a methodologically valid alternative for improving the behavior of existing metaheuristics.

The ablation study isolated the effect of the proposed temporal trajectory for β. LMOBIOINSPIRED achieved the best average rank among the four evaluated configurations and significantly outperformed the abrupt transition after Holm’s correction. However, its differences from the linear schedule and the low constant value did not remain significant, indicating that the gradual transition contributes favorably to performance, although its advantage is not uniform across all parameter-control strategies.

The additional evaluation on the 12 CEC 2022 functions retained the previously selected configuration without further tuning. LMOBIOINSPIRED again achieved the best average rank (3.0833) and obtained the lowest median fitness on four functions. However, after correction for multiple comparisons, statistical significance was retained only against LMOAIWPSO. This result provides further favorable evidence, while also showing that the improvements depend on the problem characteristics and are not uniform against all parameter-control strategies.

Transferring the mechanism to GWO demonstrated that the bio-inspired trajectory can be incorporated into the parameter *a* without modifying the fundamental operators of the algorithm. Nevertheless, GWOBIOINSPIRED did not achieve a statistically significant advantage over the original linear schedule. Therefore, this experiment demonstrates the feasibility of the transfer, but it does not establish that the mechanism generalizes directly to other metaheuristics. Its adaptation may need to consider the specific role played by the selected parameter within each algorithm.

Overall, the findings indicate that bio-inspiration, when applied to the design of adaptive parameter-control mechanisms, can yield improvements and be considered an alternative when developing or modifying metaheuristics. The absence of significant differences in some comparisons does not invalidate the approach; instead, it defines the scope of the current evidence and leaves open a potential research direction focused on the bio-inspired design of internal parameters.

Future work should examine alternative hyperparameter-tuning strategies and objective functions while preserving a global configuration and an independent separation between tuning and evaluation. Further studies should also investigate algorithm-specific adaptations of the mechanism rather than assuming direct transferability. Additional directions include extending the approach to binary and combinatorial optimization through a binary version of LMO and its evaluation on the Set Covering Problem, validating it on real-world applications such as metaheuristic-based feature selection for agricultural disease detection [42], and investigating other biological phenomena as sources of temporal trajectories for internal parameters.

## Figures and Tables

**Table 1:** Statistical Summary of the Normalized Exploration and Exploitation Indicator Values.

Algorithm	Exploration Indicator %	Std. Dev.	Exploitation Indicator %	Std. Dev.
AOA	30.716	36.102	69.284	36.102
PSA	19.763	4.974	80.237	4.974
PSO	8.635	0.820	91.365	0.820
SBOA	10.644	8.823	89.356	8.823
SCA	52.430	9.719	47.570	9.719
WOA	19.493	9.197	80.507	9.197

The normalized exploration and exploitation indicators are reported as observed mean values across iterations, independent runs, and benchmark functions.

**Table 2:** Bio-inspired principles used for the modeling of β.

Observation from Bird Landing Maneuvers	Interpretation in Optimization and Experimental Evidence
Greater freedom of movement during the initial approach	Corresponds to an early exploratory phase. The observed mean exploration indicator ranged from approximately 10% (PSO) to approximately 30% (AOA) in five of the six evaluated algorithms, whereas it exceeded 50% for SCA.
Progressive reduction of freedom of movement as the target is approached	Exploration should decrease progressively as the search advances. This implies a gradual reduction of the β value.
Increased control and precision during the final landing phase	Exploitation should dominate as the search advances. The observed mean exploitation indicator ranged from approximately 70% (AOA) to approximately 90% (PSO) in five of the six evaluated algorithms, whereas it remained below 50% for SCA.
Continuous adaptation to environmental disturbances	The search dynamics should not depend on a fixed value of β, but rather on its temporal evolution, whose trajectory is determined by previously tuned hyperparameters.
Continuous maneuvering without abrupt behavioral changes	The transition between exploration and exploitation should be smooth and progressive.

**Table 3:** Stratified partition of the benchmark functions into tuning and test sets.

Category	Tuning Functions	Test Functions
Unimodal	F1,F3,F6,F7	F2,F4,F5
Multimodal	F9,F12,F13	F8,F10,F11
Fixed-dimension multimodal	F15,F17,F18,F22,F23	F14,F16,F19,F20,F21
Total	12	11

**Table 4:** Normalized results obtained for each tuning function using the selected global configuration.

Benchmark Function	$R_{i}$	Distance Reduction
F1	$2.051105\times 10^{-6}$	99.999794890%
F3	$2.101148\times 10^{-2}$	97.898851601%
F6	$4.421499\times 10^{-6}$	99.999557850%
F7	$3.254924\times 10^{-4}$	99.967450765%
F9	$7.214392\times 10^{-2}$	92.785608261%
F12	$2.282152\times 10^{-9}$	99.999999772%
F13	$5.531855\times 10^{-11}$	99.999999994%
F15	$6.146783\times 10^{-3}$	99.385321690%
F17	$4.933671\times 10^{-5}$	99.995066329%
F18	$5.102957\times 10^{-11}$	99.999999995%
F22	$1.477342\times 10^{-5}$	99.998522658%
F23	$1.122597\times 10^{-5}$	99.998877403%

**Table 5:** First-ranked LMO variant for each test function according to the median final fitness obtained from 31 independent runs. Median values are reported to four significant digits.

Function	First-Ranked Variant	Median Final Fitness
F2	LMOBIOINSPIRED	0.7255
F4	LMOBIOINSPIRED	1.710
F5	LMOBIOINSPIRED	101.0
F8	LMOB01	−7196
F10	LMOBIOINSPIRED	0.5032
F11	LMOBIOINSPIRED	0.3579
F14	LMOQUADRATICDECAY	0.9980
F16	LMOQUADRATICDECAY	−1.032
F19	LMOBIOINSPIRED	−3.863
F20	LMOBIOINSPIRED	−3.322
F21	LMOBIOINSPIRED	−10.15

**Table 6:** Average Friedman ranks for the test functions.

Position	Variant	Average Rank
1	LMOBIOINSPIRED	1.7273
2	LMOQUADRATICDECAY	2.0909
3	LMOEXP	3.8182
4	LMOGWO	4.4545
5	LMOB01	4.6364
6	LMOB025	5.9091
7	LMOB05	6.5455
8	LMOB1	7.7273
9	LMOAIWPSO	8.0909

**Table 7:** Post-hoc Wilcoxon comparisons between LMOBIOINSPIRED and the remaining variants.

Compared Variant	W/T/L	*p*	$p_{\mathrm{Holm}}$	$r_{\mathrm{rb}}$	Significant
LMOQUADRATICDECAY	9/0/2	0.02441	0.05859	0.7576	No
LMOEXP	10/0/1	0.01855	0.05859	0.7879	No
LMOGWO	10/0/1	0.01367	0.05859	0.8182	No
LMOB01	10/0/1	0.04199	0.05859	0.6970	No
LMOB025	11/0/0	0.00098	0.00781	1.0000	Yes
LMOB05	10/0/1	0.00977	0.05859	0.8485	No
LMOB1	10/0/1	0.00977	0.05859	0.8485	No
LMOAIWPSO	10/0/1	0.00195	0.01367	0.9697	Yes

**Table 8:** Median final fitness over 31 runs on the held-out test functions. The lowest values are shown in bold.

Function	LMOBIOINSPIRED	LMOSTEP	LMOGWO	LMOB01
F2	**0.726**	62.1	3.25	5.73
F4	**1.71**	55.2	6.60	2.50
F5	**101**	$2.17\times 10^{7}$	617	364
F8	−7120	−3580	−6900	** −7200 **
F10	**0.503**	18.1	2.86	1.96
F11	**0.358**	168	1.15	1.04
F14	1.99	3.97	**0.998**	5.93
F16	** −1.03 **	** −1.03 **	** −1.03 **	** −1.03 **
F19	** −3.86 **	** −3.86 **	** −3.86 **	** −3.86 **
F20	** −3.32 **	−3.12	** −3.32 **	** −3.32 **
F21	** −10.2 **	−3.59	** −10.2 **	−5.05
Average rank	**1.5909**	3.6364	2.4091	2.3636

**Table 9:** Pairwise Wilcoxon signed-rank comparisons between LMOBIOINSPIRED and the ablated configurations. The win/tie/loss record is reported from the perspective of LMOBIOINSPIRED.

Comparison	W/T/L	*T*	*p*	$p_{\mathrm{Holm}}$	$r_{\mathrm{rb}}$
LMOBIOINSPIRED vs. LMOSTEP	9/2/0	0.0	0.003906	0.011719	1.0000
LMOBIOINSPIRED vs. LMOGWO	6/4/1	2.0	0.046875	0.093750	0.8571
LMOBIOINSPIRED vs. LMOB01	7/3/1	7.0	0.148438	0.148438	0.6111

**Table 10:** First-ranked LMO variant for each CEC 2022 test function according to the median final fitness obtained from 31 independent runs. Median values are reported to four significant digits.

Function	First-Ranked Variant	Median Final Fitness
F1	LMOBIOINSPIRED	300.0
F2	LMOB01	404.5
F3	LMOBIOINSPIRED	600.0
F4	LMOGWO	828.0
F5	LMOBIOINSPIRED	900.0
F6	LMOB01	$3.412\times 10^{4}$
F7	LMOQUADRATICDECAY	2029
F8	LMOB01	2242
F9	LMOB025	2301
F10	LMOB01	2623
F11	LMOBIOINSPIRED	2600
F12	LMOEXP	2867

**Table 11:** Average Friedman ranks for the CEC 2022 functions.

Position	Variant	Average Rank
1	LMOBIOINSPIRED	3.0833
2	LMOQUADRATICDECAY	3.5000
3	LMOEXP	3.5833
4	LMOB025	4.1667
5	LMOB01	4.2500
6	LMOGWO	4.4167
7	LMOB05	6.1667
8	LMOB1	7.9167
8	LMOAIWPSO	7.9167

**Table 12:** Post-hoc Wilcoxon comparisons between LMOBIOINSPIRED and the remaining variants on the CEC 2022 functions.

Compared Variant	W/T/L	*p*	$p_{\mathrm{Holm}}$	$r_{\mathrm{rb}}$	Significant
LMOQUADRATICDECAY	8/0/4	0.46973	1.00000	0.2564	No
LMOEXP	9/0/3	0.33936	1.00000	0.3333	No
LMOGWO	8/0/4	0.73340	1.00000	0.1282	No
LMOB01	6/0/6	0.56934	1.00000	−0.2051	No
LMOB1	11/0/1	0.00928	0.06494	0.8205	No
LMOB025	7/0/5	0.73340	1.00000	−0.1282	No
LMOAIWPSO	12/0/0	0.00049	0.00391	1.0000	Yes
LMOB05	10/0/2	0.15137	0.90820	0.4872	No

**Table 13:** Median final fitness and per-function ranks for GWO and GWOBIOINSPIRED.

Function	GWO	Rank	GWOBIOINSPIRED	Rank	Best Method
F2	0.00211	1.0	0.104	2.0	GWO
F4	0.353	1.0	2.06	2.0	GWO
F5	28.8	1.0	38.0	2.0	GWO
F8	−4040	2.0	−4630	1.0	GWOBIOINSPIRED
F10	0.00260	1.0	0.0943	2.0	GWO
F11	0.000998	1.0	0.258	2.0	GWO
F14	3.98	2.0	2.98	1.0	GWOBIOINSPIRED
F16	−1.03	1.5	−1.03	1.5	Tie
F19	−3.86	1.5	−3.86	1.5	Tie
F20	−3.32	1.5	−3.32	1.5	Tie
F21	−10.1	2.0	−10.2	1.0	GWOBIOINSPIRED

## Data Availability

The source code and the experimental artifacts supporting the findings of this study are publicly available at https://github.com/beyond-bio-inspired-algorithms/lmo-bio-inspired (accessed on 18 August 2026) (release v1.0). The repository contains the implementations of all metaheuristics and β-control variants, the benchmark and CEC 2022 function definitions, the solver and database utilities, the computation of the diversity and exploration/exploitation indicators, the Optuna tuning scripts, the ablation-study configuration, the analysis scripts, and the complete backups of the six reported experiments, each with its experimental conditions, reproduction commands, source snapshot and checksums.

## Abbreviations

The following abbreviations are used in this manuscript:

LMO	Logarithmic Mean Optimization
PSO	Particle Swarm Optimization
WOA	Whale Optimization Algorithm
SCA	Sine Cosine Algorithm
SBOA	Secretary Bird Optimization Algorithm
PSA	Pendulum Search Algorithm
TPE	Tree-structured Parzen Estimator
AOA	Arithmetic Optimization Algorithm
